# Supplementary material for: Assessing Assembly Errors in Immunoglobulin Loci: A Comprehensive Evaluation of Long-read Genome Assemblies Across Vertebrates
Source: bioRxiv. 2024 Aug 2:2024.07.19.604360. Originally published 2024 Jul 23. Preprint. [Version 2] doi: 10.1101/2024.07.19.604360 (PMC11291089; doi:10.1101/2024.07.19.604360)
Supplement: Supplement 1 [file media-1.pdf]

IGH

Class	IndividualID	LatinName	CommonName	Source	SourceLink	Haplotype Resolved	Evaluation Result	Special Condition	Curation Status
Mammalia	mApoSyl1	Apodemus sylvaticus	wood mouse	VGP	o/genomeark-all/A	No	Mismatch; Break in Primary assembly; Abnormal high coverage	alternate locus too short/not found	
Mammalia	mBalAcu1	Balaenoptera_acutorostrata	minke whale	VGP	rg/vgp-all/Balaen	No	Good	Slight mismatch	
Mammalia	mCamDro1	Camelus dromedarius	dromedary	VGP	j/genomeark-all/C	Yes	Good	Slight Mismatch	
Mammalia	mCanLor1	Canis_lupus	Greenland Wolf	VGP	e/s3/genomeark/s	No	Mismatch	Short Alternate Assembly	Missing Contig in Alternate assembly
Mammalia	mCanLor2	Canis_lupus	Greenland Wolf	VGP	e/s3/genomeark/s	Yes	Break in Primary assembly		false inversion
Mammalia	mCerEla1	Cervus_elaphus	Red Deer	VGP	s3/genomeark/spe	No	Good	Slight Mismatch; Short Alternate Assembly	
Mammalia	mChiNiv1	Chionomys nivalis	European snow vole	VGP	.io/genomeark-all	No	Good	Slight Mismatch; alternate locus too short/not found	
Mammalia	mCorTow1.0	Corynorhinus townsendii	Townsend's Big-eared Bat	CCGP	s/corynorhinus-tov	Yes	Break in Primary + Alternate assembly	2 IGH; Slight Mismatch	
Mammalia	mCynVol1	Cynocephalus volans	Philippine flying lemur	VGP	genomeark/specie	No	Mismatch	Short Alternate Assembly	Missing Contig in Alternate assembly
Mammalia	mDasNov1	Dasypus novemcinctus	nine-banded armadillo	VGP	/genomeark-all/D	Yes	Break in Primary + Alternate Assembly		
Mammalia	mDelDel1	Delphinus delphis	saddleback dolphin	VGP	ark.org/vgp-all/De	No	Mismatch	Short Alternate Assembly	
Mammalia	mDicBic1	Diceros bicornis	black rhinoceros	VGP	b.io/genomeark-a	Yes	Mismatch; Break in Primary assembly		
Mammalia	mDipMer1	Dipodomys merriami	Merriam's Kangaroo Rat	CCGP	cies/dipodomys-m	No	Good		
Mammalia	mEleMax1	Elephas maximus	Asiatic Elephant	VGP	3/genomeark/spec	No	Good	Short Alternate Assembly	
Mammalia	mEptNil1	Eptesicus nilssonii	northern bat	VGP	ark.org/vgp-all/Ept	No	Mismatch; Break in Alternate Assembly	2 IGH	
Mammalia	mEriEur2	Erinaceus europaeus	western European hedgehog	VGP	k.org/vgp-all/Erina	No	Mismatch; Break in Primary assembly	alternate locus too short/not found	
Mammalia	mEscRob2	Eschrichtius robustus	grey whale	VGP	k.org/vgp-all/Esch	No	Good		
Mammalia	mEubGla1	Eubalaena glacialis	North Atlantic right whale	VGP	rk.org/vgp-all/Eub	Yes	Good		
Mammalia	mGloMel1	Globicephala melas	long-finned pilot whale	VGP	rk.org/vgp-all/Glol	No	Good	Short Alternate Assembly	
Mammalia	mGorGor1	Gorilla_gorilla	Gorilla	T2T Primate	/s3/genomeark/sp	Yes	Good		
Mammalia	mHetBru1	Heterohyrax brucei	Yellow-spotted hyrax	VGP	.io/genomeark-all/	No	Good	Short Alternate Assembly	
Mammalia	mHipAmp2	Hippopotamus amphibius kiboko	hippopotamus	VGP	ark-curated-asser	Yes	Good	Slight Mismatch; Most MapQ 0 reads; Short Alternate Assembly	
Mammalia	mHypAmp2	Hyperoodon ampullatus	northern bottlenose whale	VGP	.org/vgp-all/Hyper	No	Good	Short Alternate Assembly	
Mammalia	mLagAlb1	Lagenorhynchus albirostris	white-beaked dolphin	VGP	rg/vgp-all/Lageno	No	Mismatch	alternate locus too short/not found	
Mammalia	mLemCat1	Lemur catta	Ring-tailed lemur	VGP	hub.io/genomeark	No	Break in Primary assembly (but also is the beginning of chrom)		
Mammalia	mLynRuf1	Lynx rufus	Bobcat	CCGP	oject.org/species/	No	Good	alternate locus too short/not found	
Mammalia	mMacEug1	Macropus eugenii	tammar wallaby	VGP	.io/genomeark-all	No	Mismatch; Break in Primary assembly	alternate locus too short/not found	
Mammalia	mManPen7	Manis pentadactyla	Chinese pangolin	VGP	io/genomeark-all/	Yes	Good	Slight Mismatch	
Mammalia	mMarMar1	Martes martes	European pine marten	VGP	eark.org/vgp-all/M	No	Mismatch	alternate locus too short/not found	
Mammalia	mMelMel3	Meles meles	European badger	VGP	hub.io/genomeark-	Yes	Good	Slight Mismatch	
Mammalia	mMesDen1	Mesoplodon densirostris	Blainville's beaked whale	VGP	org/vgp-all/Mesop	No	Good		
Mammalia	mMicCal1.0	Microtus californicus	California Vole	CCGP	/species/microtus-	Yes	Break in Primary + Alternate Assembly		
Mammalia	mMicMin1	Micromys minutus	European harvest mouse	VGP	ark.org/vgp-all/Mic	No	Mismatch	alternate locus too short/not found	
Mammalia	mMirAng1	Mirounga angustirostris	Northern Elephant Seal	CCGP	es/mirounga-angu	Yes	Break in Alternate assembly		
Mammalia	mMonDom1	Monodelphis_domestica	gray short-tailed opossum	VGP	nomeark/species/	No	Mismatch		
Mammalia	mMunRee1	Muntiacus reevesi	Reeves' muntjac	VGP	ark.org/vgp-all/Mu	No	Good		
Mammalia	mMusAve1	Muscardinus avellanarius	hazel dormouse	VGP	org/vgp-all/Musca	No	Good	alternate locus too short/not found	
Mammalia	mMusLut2	Mustela lutreola	European mink	VGP	.org/genomeark-a	No	Good		
Mammalia	mMusNiv1	Mustela nivalis	Least weasel	VGP	k.org/genomeark-a	Yes	Break in Alternate Assembly		
Mammalia	mMyoDau2	Myotis daubentonii	Daubenton's bat	VGP	ark.org/vgp-all/My	No	Mismatch	2 IGH	
Mammalia	mMyoYum1.0	Myotis yumanensis	Yuma myotis	CCGP	g/species/myotis-y	Yes	Break in Primary + Alternate Assembly	2 IGH	
Mammalia	mNeoNeb1	Neofelis_nebulosa	Clouded Leopard	VGP	3/genomeark/spec	No	Mismatch	Abnormal 2x coverage region; Short Alternate Assembly	
Mammalia	mNycCou1	Nycticebus coucang	slow loris	VGP	io/genomeark-all/I	No	Break in Alternate assembly	Short Primary Assembly	
Mammalia	mOrcOrc1	Orcinus orca	killer whale	VGP	hub.io/genomeark-	No	Good	Short Alternate Assembly	
Mammalia	mOryCun1	Oryctolagus cuniculus	rabbit	VGP	k.org/vgp-all/Oryc	No	Mismatch	alternate locus too short/not found	
Mammalia	mPanPan1	Pan paniscus	Bonobo	T2T Primate	/s3/genomeark/sp	Yes	Good		
Mammalia	mPerMan1	Peromyscus maniculatus	deer mouse	CCGP	pecies/peromyscu	No	Break in Primary Assembly	Slight Mismatch	
Mammalia	mPhoPho1	Phocoena phocoena	harbor porpoise	VGP	k.org/vgp-all/Phoc	No	Mismatch		
Mammalia	mPipPyg2	Pipistrellus pygmaeus	soprano pipistrelle	VGP	k.org/vgp-all/Pipis	No	Good	Slight Mismatch	
Mammalia	mPleAur1	Plecotus auritus	brown big-eared bat	VGP	eark.org/vgp-all/Pl	No	Mismatch; Break in Primary Assembly	2 IGH; alternate locus too short/not found	
Mammalia	mPonAbe1	Pongo_abelii	Sumatran orangutan	T2T Primate	/s3/genomeark/sp	Yes	Good		
Mammalia	mPonPyg2	Pongo_pygmaeus	Bornean orangutan	T2T Primate	3/genomeark/spec	Yes	Good		
Mammalia	mPseCra1	Pseudorca crassidens	false killer whale	VGP	k.org/vgp-all/Pseu	Yes	Good		
Mammalia	mPumCon1.1	Puma concolor	Mountain Lion	CCGP	org/species/puma-	Yes	Good		
Mammalia	mSorAra2/1	Sorex araneus	Common shrew	VGP	ub.io/genomeark-a	No	Mismatch	Abnormal hig coverage; Short Primary Assembly	
Mammalia	mSteCoe1	Stenella coeruleoalba	striped dolphin	VGP	k.org/vgp-all/Sten	No	Mismatch		
Mammalia	mTalEur1	Talpa europaea	European mole	VGP	eark.org/vgp-all/Ta	No	Mismatch; Break in Primary assembly	alternate locus too short/not found	
Mammalia	mThoBot1	Thomomys bottae	Botta's pocket gopher	CCGP	g/species/thomom	No	Good		
Mammalia	mUrsAme1	Ursus americanus	American black bear	CCGP	gov/datasets/gen	No	Good	Slight Mismatch; alternate locus too short/not found	
Mammalia	mUrsArc2	Ursus arctos	brown bear	NCBI	gov/datasets/gen	No	Good	2 IGH; Slight Mismatch	
Mammalia	mVesMur1	Vespertilio murinus	particolored bat	VGP	rk.org/vgp-all/Ves	No	Mismatch	2 IGH	
Crocodilia	rAllMis2	Alligator mississippiensis	American alligator	VGP	genomeark/specie	Yes	Break in Primary Assembly		
Testudines	rCarCar2	Caretta caretta	Loggerhead turtle	VGP	eark.org/vgp-all/C	Yes	Break in Alternate Assembly; Double Coverage in Primary Assembly	Abnormal 2x coverage region	
Testudines	rEmyOrb1	Emys orbicularis	European pond turtle	VGP	ark.org/vgp-all/Er	Yes	Break in Primary + Alternate Assembly		
Testudines	rEryReg1	Erythrolamprus reginae	royal ground snake	VGP	.org/vgp-all/Erythr	No	Mismatch; Break in Primary Assembly		
Squamata	rLiaOli1	Liasis olivaceus	olive python	VGP	eark.org/vgp-all/Li	Yes	Break in Primary assembly	Slight Mismatch	
Squamata	rLiaOli2	Liasis olivaceus	olive python	VGP	eark.org/vgp-all/Li	Yes	Break in Primary assembly	Slight Mismatch	
Testudines	rMalTer1	Malaclemys terrapin	diamondback terrapin	VGP	rk.org/vgp-all/Mala	Yes	Break in Primary assembly	Slight Mismatch	
Squamata	rPodCre2	Podarcis cretensis	cretan wall lizard	VGP	ark.org/vgp-all/Poc	No	Break in Primary assembly	Slight Mismatch; alternate locus too short/not found	

Class	IndividualID	LatinName	CommonName	Source	SourceLink	Haplotype Resolved	Evaluation Result	Special Condition	Curation Status
Squamata	rPodRaf1	Podarcis raffonei	Aeolian wall lizard	VGP	ark.org/vgp-all/Po	No	Good	alternate locus too short/not found	
Squamata	rRhiFlo1	Rhineura floridana	Florida worm lizard	VGP	ark.org/vgp-all/Rhi	Yes	Break in Primary + Alternate Assembly	Slight Mismatch; Short Alternate Assembly	
Squamata	rVipLat1	Vipera latastei	snub-nosed viper	VGP	eark.org/vgp-all/V	No	Mismatch; Break in Primary Assembly	alternate locus too short/not found	
Squamata	rVipUrs1	Vipera ursinii	Hungarian meadow viper	VGP	neark.org/vgp-all/V	No	Mismatch; Break in Primary Assembly	alternate locus too short/not found	
Squamata	rZooViv1	Zootoca vivipara	common lizard	VGP	ark.org/vgp-all/Zo	No	Mismatch	alternate locus too short/not found	

IGK

Class	IndividualID	LatinName	CommonName	Source	SourceLink	Haplotype Resolved	Evaluation Result	Special Condition
Mammalia	mApoSyl1	Apodemus sylvaticus	wood mouse	VGP	o/genomeark-all/A	No	Mismatch; Break in Primary + Alternate Assembly	Short Alternate Assembly
Mammalia	mBalAcu1	Balaenoptera_acutorostrata	minke whale	VGP	rg/vgp-all/Balaen	No	Good	alternate locus too short/not found
Mammalia	mCamDro1	Camelus dromedarius	dromedary	VGP	g/genomeark-all/C	Yes	Good	
Mammalia	mCanLor1	Canis_lupus	Greenland Wolf	VGP	e/s3/genomeark/s	No	Good	
Mammalia	mCanLor2	Canis_lupus	Greenland Wolf	VGP	e/s3/genomeark/s	Yes	Good	All MapQ 0 reads
Mammalia	mCerEla1	Cervus_elaphus	Red Deer	VGP	s3/genomeark/spe	No	Good	
Mammalia	mChiNiv1	Chionomys nivalis	European snow vole	VGP	.io/genomeark-all	No	Mismatch	Short Alternate Assembly
Mammalia	mDasNov1	Dasypus novemcinctus	nine-banded armadillo	VGP	/genomeark-all/D	Yes	Mismatch; Break in Primary + Alternate Assembly	
Mammalia	mDelDel1	Delphinus delphis	saddleback dolphin	VGP	ark.org/vgp-all/De	No	Good	
Mammalia	mDicBic1	Diceros bicornis	black rhinoceros	VGP	b.io/genomeark-a	Yes	Good	
Mammalia	mDipMer1	Dipodomys merriami	Merriam's Kangaroo Rat	CCGP	cies/dipodomys-m	No	Good	3 IGK
Mammalia	mEleMax1	Elephas maximus	Asiatic Elephant	VGP	3/genomeark/spec	No	Good	
Mammalia	mEriEur2	Erinaceus europaeus	western European hedgehog	VGP	k.org/vgp-all/Erina	No	Good	alternate locus too short/not found
Mammalia	mEscRob2	Eschrichtius robustus	grey whale	VGP	k.org/vgp-all/Esch	No	Good	
Mammalia	mEubGla1	Eubalaena glacialis	North Atlantic right whale	VGP	rk.org/vgp-all/Eub	Yes	Good	
Mammalia	mGloMel1	Globicephala melas	long-finned pilot whale	VGP	rk.org/vgp-all/Glol	No	Good	alternate locus too short/not found
Mammalia	mGorGor1	Gorilla_gorilla	Gorilla	T2T Primate	/s3/genomeark/sp	Yes	Good	
Mammalia	mHetBru1	Heterohyrax brucei	Yellow-spotted hyrax	VGP	.io/genomeark-all/	No	Good	alternate locus too short/not found
Mammalia	mHipAmp2	Hippopotamus amphibius kiboko	hippopotamus	VGP	ark-curated-asser	Yes	Good	Slight Mismatch
Mammalia	mHypAmp2	Hyperoodon ampullatus	northern bottlenose whale	VGP	.org/vgp-all/Hyper	No	Mismatch	
Mammalia	mLagAlb1	Lagenorhynchus albirostris	white-beaked dolphin	VGP	rg/vgp-all/Lageno	No	Good	
Mammalia	mLemCat1	Lemur catta	Ring-tailed lemur	VGP	hub.io/genomeark	No	Good	
Mammalia	mLynRuf1	Lynx rufus	Bobcat	CCGP	oject.org/species/	No	Good	Short Alternate Assembly
Mammalia	mMacEug1	Macropus eugenii	tammar wallaby	VGP	.io/genomeark-all	No	Mismatch	alternate locus too short/not found
Mammalia	mManPen7	Manis pentadactyla	Chinese pangolin	VGP	io/genomeark-all/	Yes	Break in Alternate Assembly	
Mammalia	mMarMar1	Martes martes	European pine marten	VGP	eark.org/vgp-all/M	No	Mismatch	alternate locus too short/not found
Mammalia	mMelMel3	Meles meles	European badger	VGP	hub.io/genomeark	Yes	Good	All MapQ 0 reads
Mammalia	mMesDen1	Mesoplodon densirostris	Blainville's beaked whale	VGP	org/vgp-all/Mesop	No	Good	
Mammalia	mMicCal1.0	Microtus californicus	California Vole	CCGP	/species/microtus-	Yes	Good	
Mammalia	mMicMin1	Micromys minutus	European harvest mouse	VGP	ark.org/vgp-all/Mic	No	Mismatch	alternate locus too short/not found
Mammalia	mMirAng1	Mirounga angustirostris	Northern Elephant Seal	CCGP	es/mirounga-angu	Yes	Good	All MapQ 0 reads
Mammalia	mMonDom1	Monodelphis_domestica	gray short-tailed opossum	VGP	enomeark/species/	No	Mismatch; Break in Primary Assembly	alternate locus too short/not found
Mammalia	mMunRee1	Muntiacus reevesi	Reeves' muntjac	VGP	ark.org/vgp-all/Mu	No	Good	
Mammalia	mMusAve1	Muscardinus avellanarius	hazel dormouse	VGP	org/vgp-all/Musca	No	Good	Short Alternate Assembly
Mammalia	mMusLut2	Mustela lutreola	European mink	VGP	.org/genomeark-a	No	Good	Short Alternate Assembly
Mammalia	mMusNiv1	Mustela nivalis	Least weasel	VGP	.org/genomeark-a	Yes	Break in Alternate Assembly	Most MapQ 0 reads
Mammalia	mNeoNeb1	Neofelis_nebulosa	Clouded Leopard	VGP	3/genomeark/spec	No	Good	
Mammalia	mNycCou1	Nycticebus coucang	slow loris	VGP	io/genomeark-all/	No	Good	
Mammalia	mOrcOrc1	Orcinus orca	killer whale	VGP	hub.io/genomeark	No	Good	alternate locus too short/not found
Mammalia	mOryCun1	Oryctolagus cuniculus	rabbit	VGP	k.org/vgp-all/Oryc	No	Mismatch	alternate locus too short/not found; abnormal 2x coverage
Mammalia	mPanPan1	Pan paniscus	Bonobo	T2T Primate	/s3/genomeark/sp	Yes	Good	
Mammalia	mPerMan1	Peromyscus maniculatus	deer mouse	CCGP	pecies/peromyscu	No	Break in Alternate Assembly	Short Primary Assembly
Mammalia	mPhoPho1	Phocoena phocoena	harbor porpoise	VGP	rk.org/vgp-all/Pho	No	Good	
Mammalia	mPonAbe1	Pongo_abelii	Sumatran orangutan	T2T Primate	e/s3/genomeark/sp	Yes	Good	
Mammalia	mPonPyg2	Pongo_pygmaeus	Bornean orangutan	T2T Primate	3/genomeark/spec	Yes	Good	
Mammalia	mPseCra1	Pseudorca crassidens	false killer whale	VGP	k.org/vgp-all/Pseu	Yes	Good	
Mammalia	mPumCon1.1	Puma concolor	Mountain Lion	CCGP	org/species/puma	Yes	Good	
Mammalia	mSorAra2/1	Sorex araneus	Common shrew	VGP	ub.io/genomeark-a	No	Mismatch; Break in Primary Assembly	HUGE BREAK , Short Alternate Assembly
Mammalia	mSteCoe1	Stenella coeruleoalba	striped dolphin	VGP	k.org/vgp-all/Sten	No	Good	
Mammalia	mTalEur1	Talpa europaea	European mole	VGP	eark.org/vgp-all/Ta	No	Good	
Mammalia	mThoBot1	Thomomys bottae	Botta's pocket gopher	CCGP	g/species/thomom	No	Good	Short Primary Assembly
Mammalia	mUrsAme1	Ursus americanus	American black bear	CCGP	gov/datasets/gen	No	Good	alternate locus too short/not found
Mammalia	mUrsArc2	Ursus arctos	brown bear	NCBI	gov/datasets/gen	No	Good	
Crocodilia	rAllMis2	Alligator mississippiensis	American alligator	VGP	genomeark/specie	Yes	Good	Most MapQ 0 reads
Testudines	rCarCar2	Caretta caretta	Loggerhead turtle	VGP	eark.org/vgp-all/C	Yes	Good	
Testudines	rEmyOrb1	Emys orbicularis	European pond turtle	VGP	ark.org/vgp-all/Er	Yes	Good	Slight Mismatch; Short Primary Assembly
Testudines	rMalTer1	Malaclemys terrapin	diamondback terrapin	VGP	rk.org/vgp-all/Mal	Yes	Good	Short Primary Assembly

IGL

Class	IndividualID	LatinName	CommonName	Source	SourceLink	Haplotype Resolved	Evaluation Result	Special Condition
Mammalia	mApoSyl1	Apodemus sylvaticus	wood mouse	VGP	o/genomeark-all/A	No	Mismatch; Break in Primary Assembly	alternate locus too short/not found
Mammalia	mBalAcu1	Balaenoptera_acutorostrata	minke whale	VGP	rg/vgp-all/Balaen	No	Good	
Mammalia	mCamDro1	Camelus dromedarius	dromedary	VGP	g/genomeark-all/C	Yes	Good	
Mammalia	mCanLor1	Canis_lupus	Greenland Wolf	VGP	e/s3/genomeark/s	No	Mismatch; Break in Primary Assembly	alternate locus too short/not found
Mammalia	mCanLor2	Canis_lupus	Greenland Wolf	VGP	e/s3/genomeark/s	Yes	Break in Primary + Alternate Assembly	
Mammalia	mCerEla1	Cervus_elaphus	Red Deer	VGP	s3/genomeark/spe	No	Good	Short Alternate Assembly
Mammalia	mCorTow1.0	Corynorhinus townsendii	Townsend's Big-eared Bat	CCGP	s/corynorhinus-tov	Yes	Good	Slight Mismatch
Mammalia	mCynVol1	Cynocephalus volans	Philiphine flying lemur	VGP	genomeark/specie	No	Mismatch	alternate locus too short/not found
Mammalia	mDasNov1	Dasypus novemcinctus	nine-banded armadillo	VGP	/genomeark-all/D	Yes	Mismatch; Break in Primary + Alternate Assembly	
Mammalia	mDelDel1	Delphinus delphis	saddleback dolphin	VGP	ark.org/vgp-all/De	No	Mismatch	
Mammalia	mDicBic1	Diceros bicornis	black rhinoceros	VGP	b.io/genomeark-a	Yes	Break in Alternate Assembly	Slight Mismatch
Mammalia	mEleMax1	Elephas maximus	Asiatic Elephant	VGP	3/genomeark/spec	No	Good	
Mammalia	mEptNil1	Eptesicus nilssonii	northern bat	VGP	ark.org/vgp-all/Ept	No	Good	Slight Mismatch
Mammalia	mEriEur2	Erinaceus europaeus	western European hedgehog	VGP	k.org/vgp-all/Erina	No	Mismatch; Break in Primary Assembly	alternate locus too short/not found
Mammalia	mEscRob2	Eschrichtius robustus	grey whale	VGP	k.org/vgp-all/Esch	No	Good	
Mammalia	mEubGla1	Eubalaena glacialis	North Atlantic right whale	VGP	rk.org/vgp-all/Eub	Yes	Good	All MapQ 0 reads
Mammalia	mGloMel1	Globicephala melas	long-finned pilot whale	VGP	rk.org/vgp-all/Glol	No	Mismatch; Break in Primary Assembly	alternate locus too short/not found
Mammalia	mGorGor1	Gorilla_gorilla	Gorilla	T2T Primate	/s3/genomeark/sp	Yes	Good	
Mammalia	mHetBru1	Heterohyrax brucei	Yellow-spotted hyrax	VGP	.io/genomeark-all/	No	Mismatch	
Mammalia	mHipAmp2	Hippopotamus amphibius kiboko	hippopotamus	VGP	ark-curated-asser	Yes	Break in Alternate Assembly	All MapQ 0 reads
Mammalia	mHypAmp2	Hyperoodon ampullatus	northern bottlenose whale	VGP	.org/vgp-all/Hyper	No	Good	Short Alternate Assembly
Mammalia	mLagAlb1	Lagenorhynchus albirostris	white-beaked dolphin	VGP	rg/vgp-all/Lageno	No	Mismatch	alternate locus too short/not found
Mammalia	mLemCat1	Lemur catta	Ring-tailed lemur	VGP	nub.io/genomeark	No	Break in Primary + Alternate Assembly	Slight Mismatch
Mammalia	mLynRuf1	Lynx rufus	Bobcat	CCGP	oject.org/species/	No	Good	Slight Mismatch; alternate locus too short/not found
Mammalia	mMacEug1	Macropus eugenii	tammar wallaby	VGP	.io/genomeark-all	No	Break in Primary Assembly	alternate locus too short/not found
Mammalia	mManPen7	Manis pentadactyla	Chinese pangolin	VGP	io/genomeark-all/	Yes	Good	All MapQ 0 reads
Mammalia	mMarMar1	Martes martes	European pine marten	VGP	eark.org/vgp-all/M	No	Mismatch	alternate locus too short/not found
Mammalia	mMelMel3	Meles meles	European badger	VGP	nub.io/genomeark	Yes	Good	Most MapQ 0 reads
Mammalia	mMesDen1	Mesoplodon densirostris	Blainville's beaked whale	VGP	org/vgp-all/Mesop	No	Good	
Mammalia	mMicCal1.0	Microtus californicus	California Vole	CCGP	/species/microtus-	Yes	Good	Short Primary Assembly
Mammalia	mMicMin1	Micromys minutus	European harvest mouse	VGP	ark.org/vgp-all/Mic	No	Mismatch; Break in Primary Assembly	alternate locus too short/not found
Mammalia	mMirAng1	Mirounga angustirostris	Northern Elephant Seal	CCGP	es/mirounga-angu	Yes	Break in Primary + Alternate Assembly	2 IGL
Mammalia	mMonDom1	Monodelphis_domestica	gray short-tailed opossum	VGP	nomeark/species/	No	Break in Primary Assembly	alternate locus too short/not found
Mammalia	mMunRee1	Muntiacus reevesi	Reeves' muntjac	VGP	ark.org/vgp-all/Mu	No	Good	Slight Mismatch; alternate locus too short/not found
Mammalia	mMusAve1	Muscardinus avellanarius	hazel dormouse	VGP	org/vgp-all/Musca	No	Mismatch	alternate locus too short/not found
Mammalia	mMusLut2	Mustela lutreola	European mink	VGP	.org/genomeark-a	No	Good	Slight Mismatch; Short Alternate Assembly
Mammalia	mMusNiv1	Mustela nivalis	Least weasel	VGP	.org/genomeark-a	Yes	Mismatch; Break in Primary + Alternate Assembly	
Mammalia	mMyoDau2	Myotis daubentonii	Daubenton's bat	VGP	ark.org/vgp-all/My	No	Mismatch	alternate locus too short/not found
Mammalia	mMyoYum1.0	Myotis yumanensis	Yuma myotis	CCGP	g/species/myotis-	Yes	Good	Short Primary Assembly
Mammalia	mNeoNeb1	Neofelis_nebulosa	Clouded Leopard	VGP	3/genomeark/spec	No	Good	alternate locus too short/not found
Mammalia	mNycCou1	Nycticebus coucang	slow loris	VGP	io/genomeark-all/	No	Mismatch; Break in Primary Assembly	extremly abnormal coverage in Alternate Assembly
Mammalia	mOrcOrc1	Orcinus orca	killer whale	VGP	nub.io/genomeark	No	Good	alternate locus too short/not found
Mammalia	mOryCun1	Oryctolagus cuniculus	rabbit	VGP	k.org/vgp-all/Oryc	No	Good	alternate locus too short/not found
Mammalia	mPanPan1	Pan paniscus	Bonobo	T2T Primate	/s3/genomeark/sp	Yes	Good	
Mammalia	mPerMan1	Peromyscus maniculatus	deer mouse	CCGP	pecies/peromysc	No	Good	Slight Mismatch; Short Alternate Assembly
Mammalia	mPhoPho1	Phocoena phocoena	harbor porpoise	VGP	k.org/vgp-all/Phoc	No	Mismatch	Short Alternate Assembly
Mammalia	mPipPyg2	Pipistrellus pygmaeus	soprano pipistrelle	VGP	k.org/vgp-all/Pipis	No	Good	
Mammalia	mPonAbe1	Pongo_abelii	Sumatran orangutan	T2T Primate	e/s3/genomeark/sp	Yes	Good	
Mammalia	mPonPyg2	Pongo_pygmaeus	Bornean orangutan	T2T Primate	3/genomeark/spec	Yes	Good	
Mammalia	mPseCra1	Pseudorca crassidens	false killer whale	VGP	k.org/vgp-all/Pseu	Yes	Good	
Mammalia	mPumCon1.1	Puma concolor	Mountain Lion	CCGP	org/species/puma	Yes	Good	
Mammalia	mSorAra2/1	Sorex araneus	Common shrew	VGP	ub.io/genomeark-a	No	Mismatch; Break in Primary Assembly	Short Alternate Assembly
Mammalia	mSteCoe1	Stenella coeruleoalba	striped dolphin	VGP	k.org/vgp-all/Sten	No	Mismatch	Short Alternate Assembly
Mammalia	mTalEur1	Talpa europaea	European mole	VGP	eark.org/vgp-all/Ta	No	Break in Primary Assembly	alternate locus too short/not found
Mammalia	mUrsAme1	Ursus americanus	American black bear	CCGP	gov/datasets/gen	No	Break in Primary Assembly	2 IGL but on same chrom
Mammalia	mUrsArc2	Ursus arctos	brown bear	NCBI	gov/datasets/gen	No	Mismatch	
Mammalia	mVesMur1	Vespertilio murinus	particolored bat	VGP	rk.org/vgp-all/Ves	No	Good	
Crocodilia	rAllMis2	Alligator mississippiensis	American alligator	VGP	genomeark/specie	Yes	Good	
Testudines	rCarCar2	Caretta caretta	Loggerhead turtle	VGP	eark.org/vgp-all/C	Yes	Break in Primary Assembly	Slight Mismatch
Testudines	rEmyOrb1	Emys orbicularis	European pond turtle	VGP	ark.org/vgp-all/Er	Yes	Good	
Testudines	rMalTer1	Malaclemys terrapin	diamondback terrapin	VGP	rk.org/vgp-all/Mala	Yes	Good	
