# Supplementary material for: Assessing Assembly Errors in Immunoglobulin Loci: A Comprehensive Evaluation of Long-read Genome Assemblies Across Vertebrates
Source: bioRxiv. 2024 Aug 2:2024.07.19.604360. Originally published 2024 Jul 23. Preprint. [Version 2] doi: 10.1101/2024.07.19.604360 (PMC11291089; doi:10.1101/2024.07.19.604360)
Supplement: 1 [file NIHPP2024.07.19.604360V2-supplement-1.pdf]

bioRxiv preprint doi: https://doi.org/10.1101/2024.07.19.604360; this version posted August 2, 2024. The copyright holder for this preprint (which was not certified by peer review) is the author/funder, who has granted bioRxiv a license to display the preprint in perpetuity. It is made available under aCC-BY 4.0 International license.

Supplementary Information

Simulation

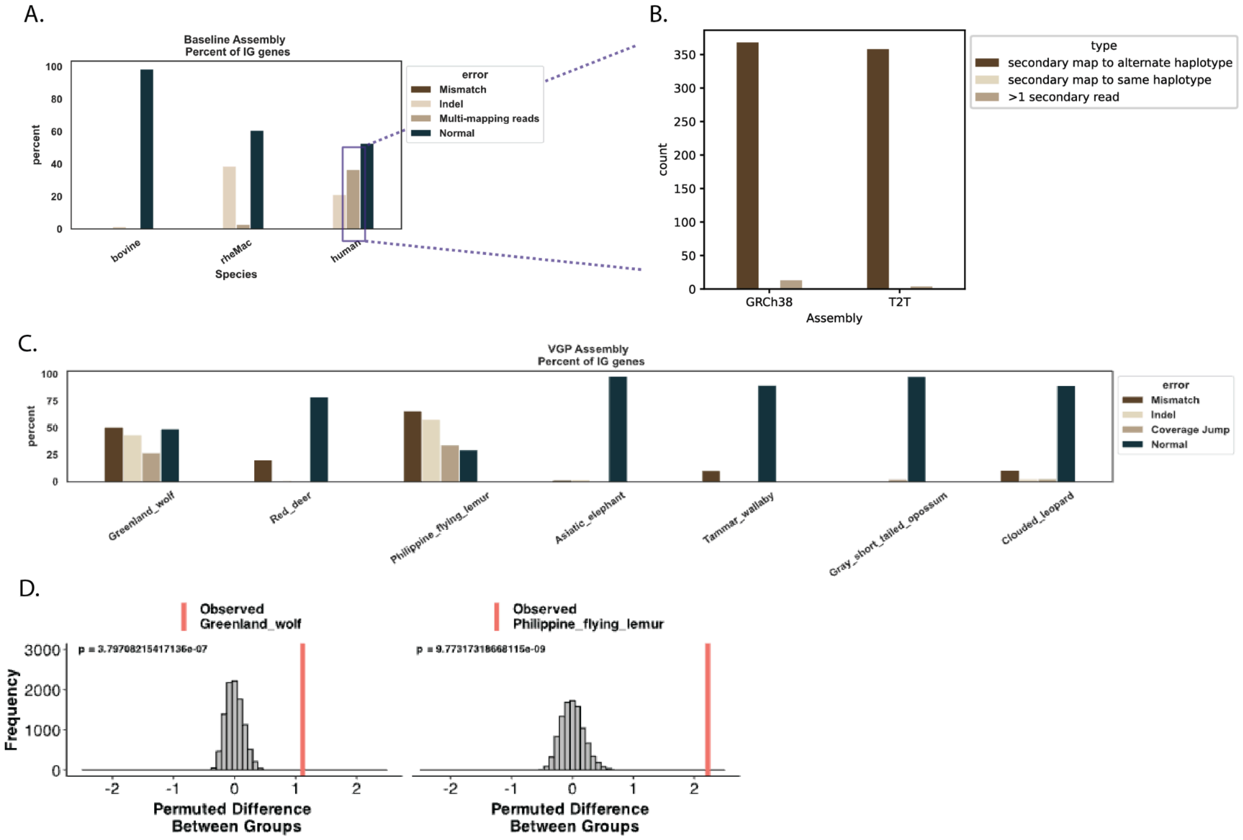

Supplementary Figure 1. Benchmarking Assembly Errors Using Simulated HiFi reads from Human, Bovine, and Rhesus assembly. All genomes are diploid, with the human genome created as a combination of the GRCh38 and T2T assemblies. A. Percent of IG genes displaying each type of alignment error within human, bovine, and rhesus assemblies using simulated reads. B. Multi-mapped reads in the human assembly were found due to low heterozygosity between the two haplotypes. C. Percent of IG genes displaying each type of alignment error within eight VGP assemblies using real sequencing reads. D. A permutation test was used to evaluate whether the observed errors in the VGP genomes were consistent with those of the reference species with simulated reads.

bioRxiv preprint doi: https://doi.org/10.1101/2024.07.19.604360; this version posted August 2, 2024. The copyright holder for this preprint (which was not certified by peer review) is the author/funder, who has granted bioRxiv a license to display the preprint in perpetuity. It is made available under aCC-BY 4.0 International license.

Breaks in Coverage

For breaks with consecutive 'N' reference sequences, which represent unknown bases, these are often linked to complex genomic structures or repetitive elements. This includes cases with one or two reads mapped, where reads may still align if the surrounding sequences provide sufficient context for alignment algorithms to operate despite uncertainties. It also covers breaks with zero reads aligned, referred as gaps in this paper, indicating completely unsequenced or unassembled regions.

In contrast, for non-'N' reference sequences, breaks with fewer than two (non zero) reads mapped suggest regions with sparse sequencing data, potentially highlighting unsupported sequences that might reflect errors in the assembly. Breaks with no reads aligned point to more significant data gaps, where the reference sequence is known but completely lacks sequencing support, providing stronger evidence of possible assembly inaccuracies.

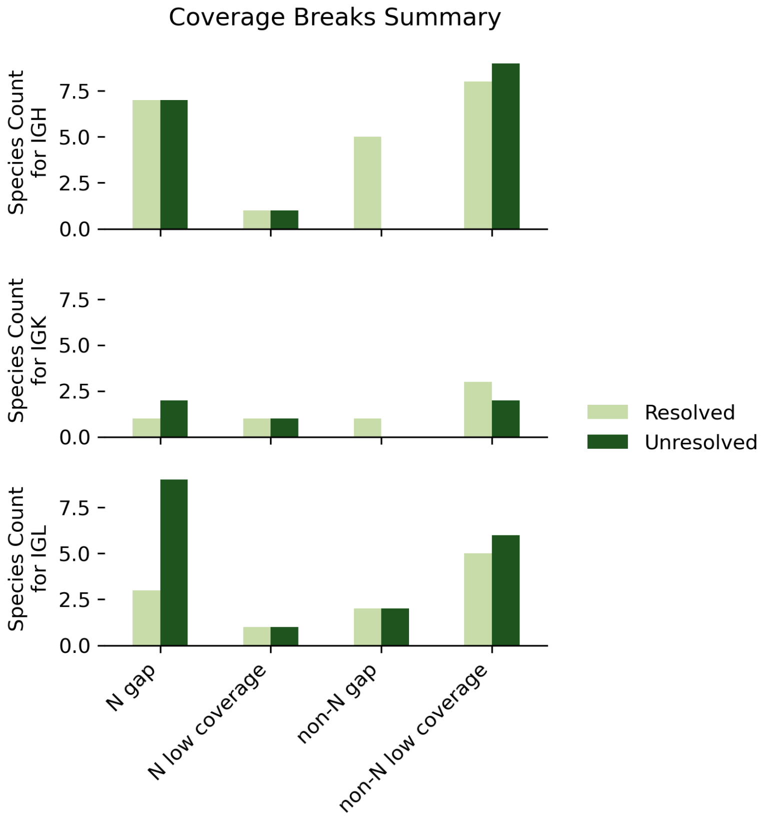

Supplementary Figure 2. Breakdown of Assembly Break Types Across Species in IGH, IGK and IGL loci. This figure displays the frequency of four distinct types of assembly breaks across various species within the IGH, IGK, and IGL loci. Break types are categorized as follows: N low coverage (fewer than two reads mapped in regions with consecutive 'N' reference sequences); N gap (zero reads mapped in regions with consecutive 'N' reference sequences); non-'N' low coverage (fewer than two reads mapped in regions with non-'N' reference sequence); and non-N gap (no reads mapped in regions with non-'N' reference sequence). Each bar is color-coded to indicate whether the species' assemblies are haplotype-resolved.

bioRxiv preprint doi: https://doi.org/10.1101/2024.07.19.604360; this version posted August 2, 2024. The copyright holder for this preprint (which was not certified by peer review) is the author/funder, who has granted bioRxiv a license to display the preprint in perpetuity. It is made available under aCC-BY 4.0 International license.

Case Studies Supplementary

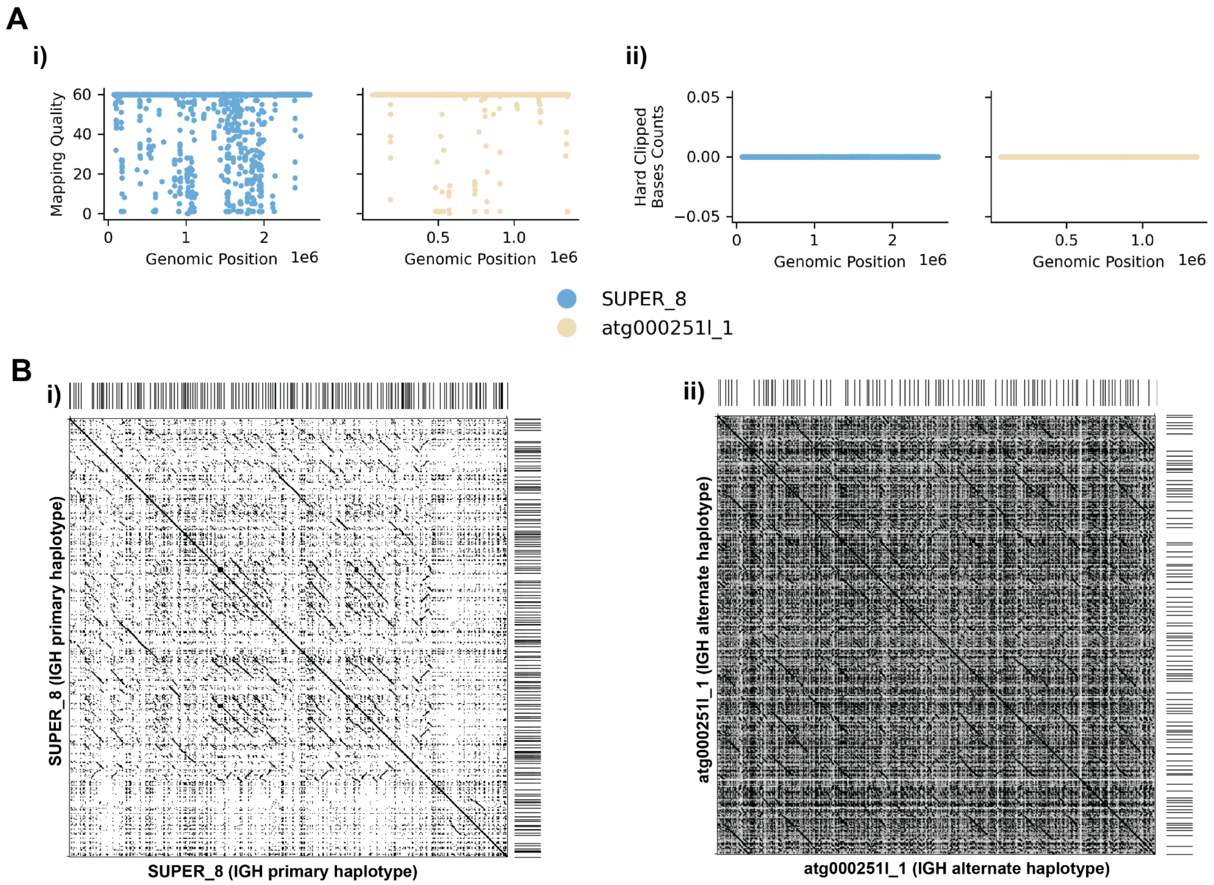

Supplementary Figure 3. Additional Analysis of IGH Locus Assembly Errors in Greenland wolf (*C. lupus*) individual 1. A. Summary statistics of the read alignment situation are depicted, showing i) mapping quality across IGH loci for both haplotypes, with blue representing the primary assembly and yellow the alternate. ii) count of hard clipped bases. B. Dotplots comparing gene locations and alignments are shown for i) primary vs primary and ii) alternate vs alternate haplotypes.

bioRxiv preprint doi: https://doi.org/10.1101/2024.07.19.604360; this version posted August 2, 2024. The copyright holder for this preprint (which was not certified by peer review) is the author/funder, who has granted bioRxiv a license to display the preprint in perpetuity. It is made available under aCC-BY 4.0 International license.

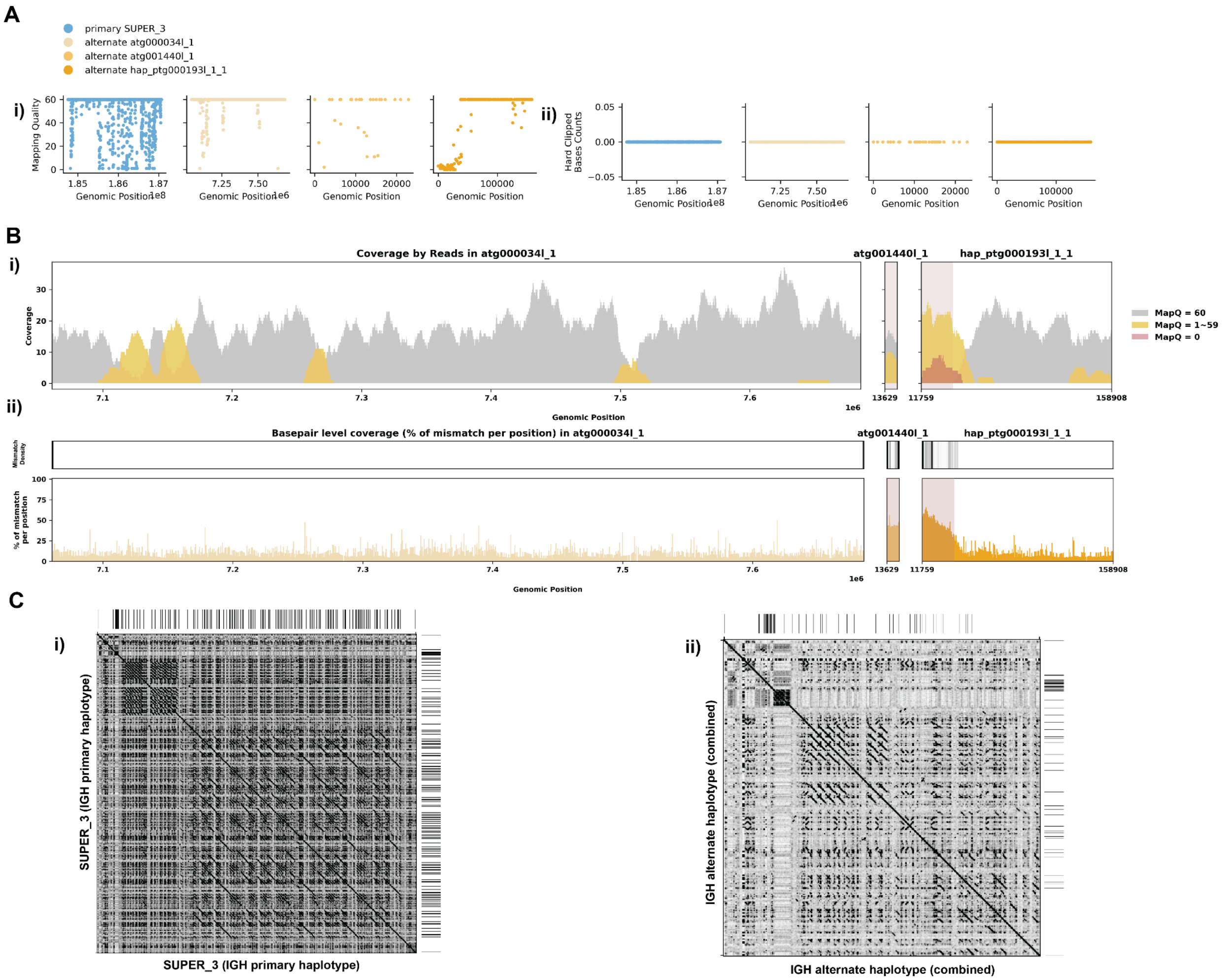

Supplementary Figure 4. Additional Analysis of IGH Locus Assembly Errors in Philippine Flying Lemur (*C. volans*). A. Summary statistics of the read alignment situation are depicted, showing i) mapping quality across IGH loci for both haplotypes, with blue representing the primary assembly and yellow the alternate. ii) count of hard clipped bases. B. A detailed analysis of alignment mismatch in the alternate IGH haplotype includes i) read coverage across the entire IGH loci, color-coded by mapping quality, and ii) *basepair-oriented* mismatch rate, a heatmap above indicating the frequency of high mismatch rate base pairs, with darker colors denoting more frequent occurrences. Light red highlights positions covered by ≥5 reads with an error rate >1%, and purple bars indicate coverage breaks (coverage ≤2). C. Dotplots comparing gene locations and alignments are shown for i) primary vs primary and ii) alternate vs alternate haplotypes.

bioRxiv preprint doi: https://doi.org/10.1101/2024.07.19.604360; this version posted August 2, 2024. The copyright holder for this preprint (which was not certified by peer review) is the author/funder, who has granted bioRxiv a license to display the preprint in perpetuity. It is made available under aCC-BY 4.0 International license.

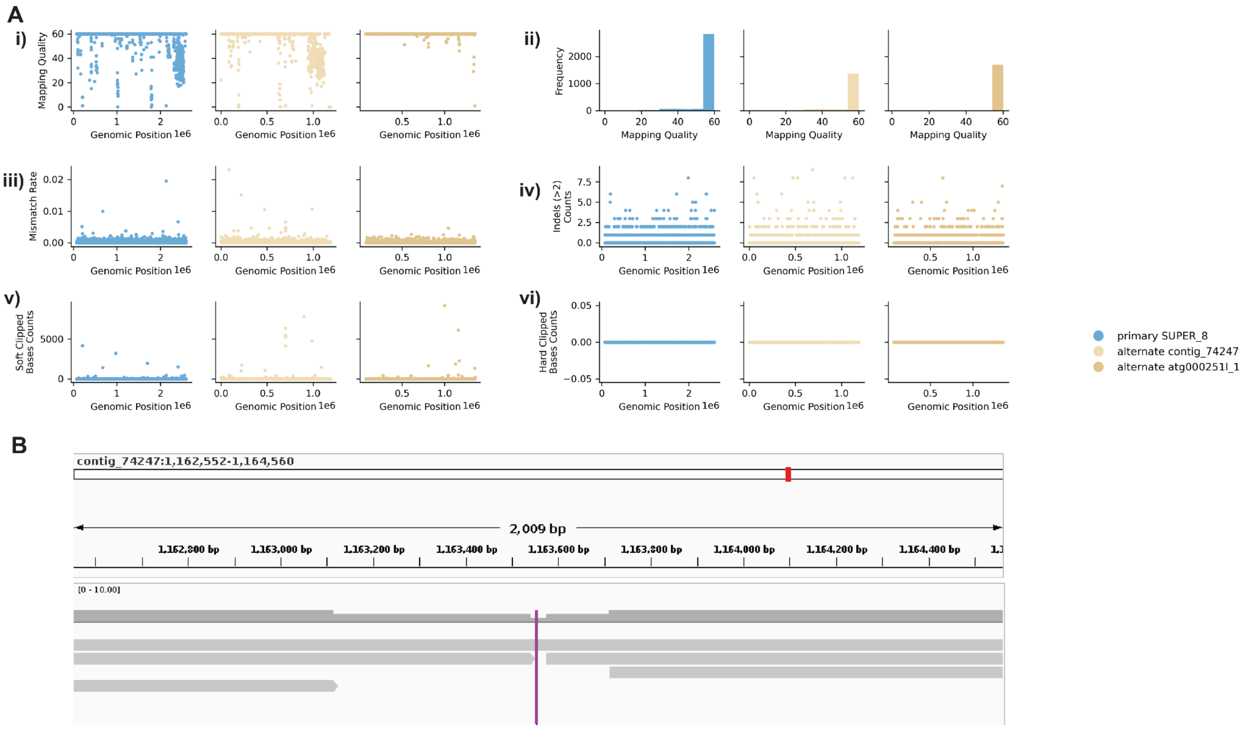

Supplementary Figure 5. Additional Reassembly Analysis of IGH Locus Assembly Errors in *C. lupus* individual 1. A. Summary statistics of the read alignment are significantly improved. Blue represents the primary assembly and yellow the alternate. i) The plots illustrate the read mapping quality across the IGH loci, ii) read mapping quality distribution, iii) mismatch rates of reads, and iv) number of indels (consecutive length > 2bps), v) count of soft clipped bases, vi) count of hard clipped bases, for both haplotypes across IGH loci. B. IGV screenshot of the low coverage region in contig “74247”, purple indicate where the break in coverage is.

bioRxiv preprint doi: https://doi.org/10.1101/2024.07.19.604360; this version posted August 2, 2024. The copyright holder for this preprint (which was not certified by peer review) is the author/funder, who has granted bioRxiv a license to display the preprint in perpetuity. It is made available under aCC-BY 4.0 International license.

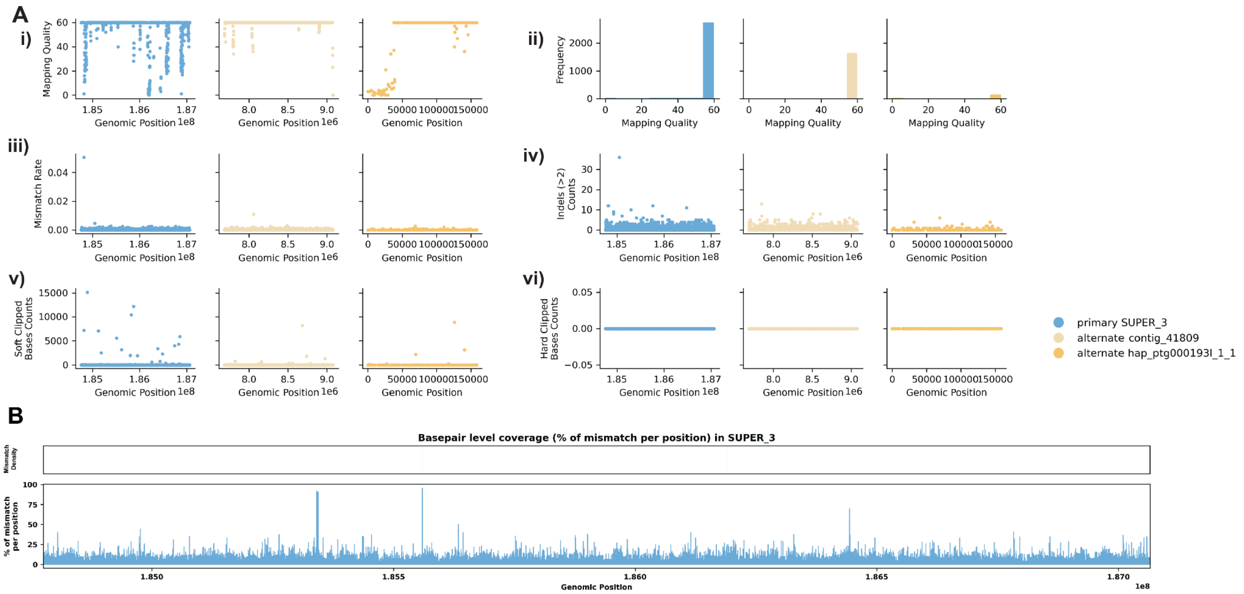

Supplementary Figure 6. Additional Reassembly Analysis of IGH Locus Assembly Errors in *C. volans*. A. Summary statistics of the read alignment are significantly improved. Blue represents the primary assembly and yellow the alternate. i) The plots illustrate the read mapping quality across the IGH loci, ii) read mapping quality distribution, iii) mismatch rates of reads, and iv) number of indels (consecutive length > 2bps), v) count of soft clipped bases, vi) count of hard clipped bases, for both haplotypes across IGH loci. B. A detailed analysis of alignment mismatch in the primary IGH haplotype from *basepair-oriented* view, a heatmap above indicating the frequency of high mismatch rate base pairs, with darker colors denoting more frequent occurrences. Purple bars indicate coverage breaks (coverage ≤2).

bioRxiv preprint doi: https://doi.org/10.1101/2024.07.19.604360; this version posted August 2, 2024. The copyright holder for this preprint (which was not certified by peer review) is the author/funder, who has granted bioRxiv a license to display the preprint in perpetuity. It is made available under aCC-BY 4.0 International license.

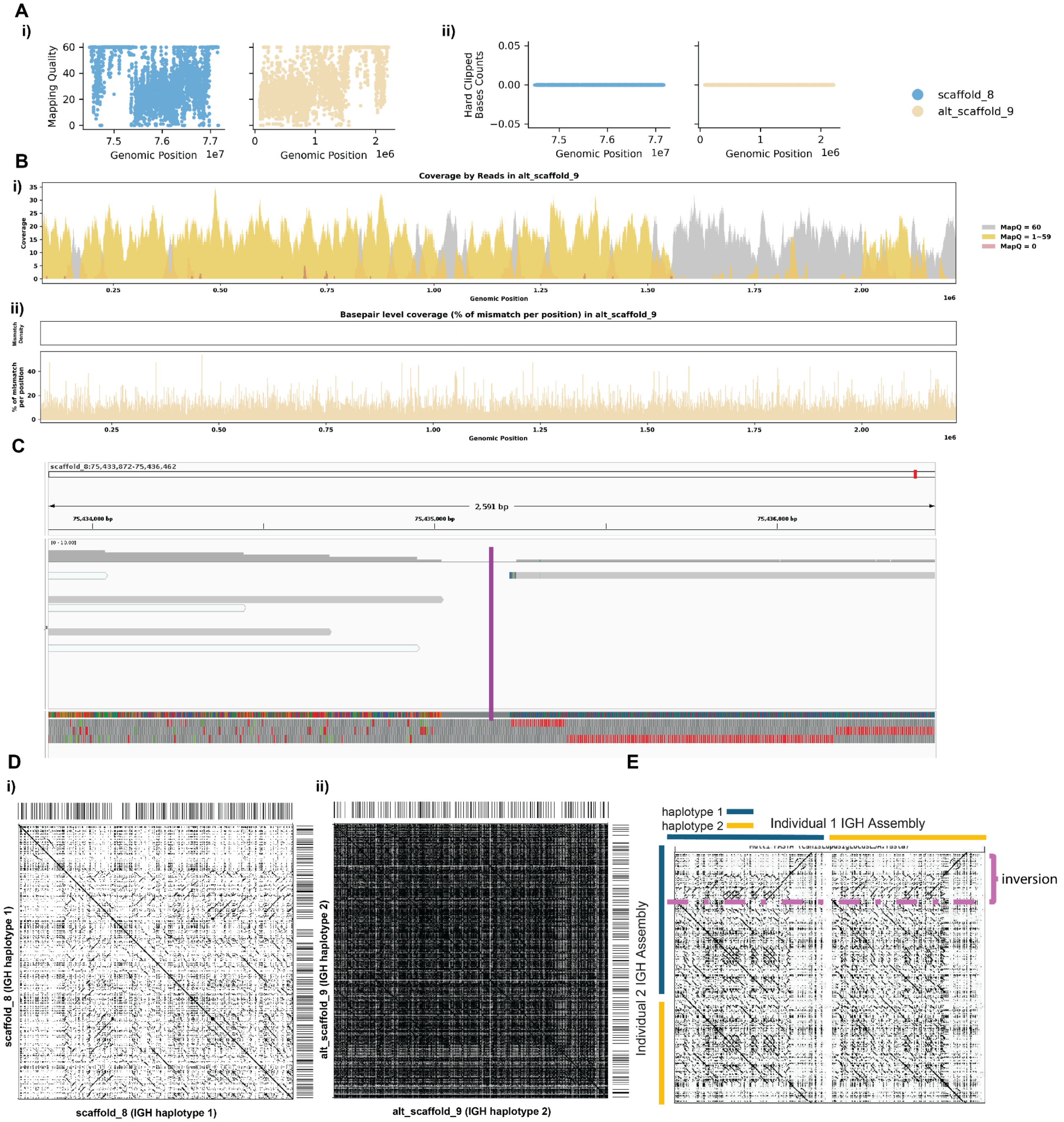

Supplementary Figure 7. Additional Detailed Analysis of IGH Locus Assembly Errors in Greenland Wolf (*C. lupus*) individual 2. A. Summary statistics of the read alignment situation are depicted, showing i) mapping quality across IGH loci for both haplotypes, with blue representing the primary assembly and yellow the alternate. ii) count of hard clipped bases. B. A detailed analysis of alignment mismatch in the alternate IGH haplotype includes i) read coverage across the entire IGH loci, color-coded by mapping quality, and ii) *basepair-oriented* mismatch rate, a heatmap above indicating the frequency of high mismatch rate base pairs, with darker colors denoting more frequent occurrences. Light red highlights positions covered by ≥5 reads with an error rate >1%, and purple bars indicate coverage breaks (coverage ≤2). C. IGV screenshot of the break in coverage D. Dotplots comparing gene locations and alignments are shown for i) primary vs primary and ii) alternate vs alternate haplotypes. E. Dotplots comparing

bioRxiv preprint doi: https://doi.org/10.1101/2024.07.19.604360; this version posted August 2, 2024. The copyright holder for this preprint (which was not certified by peer review) is the author/funder, who has granted bioRxiv a license to display the preprint in perpetuity. It is made available under aCC-BY 4.0 International license.

C. lupus individual 1 assembly vs individual 2 assembly. Purple dashed line indicate the inversion observed.

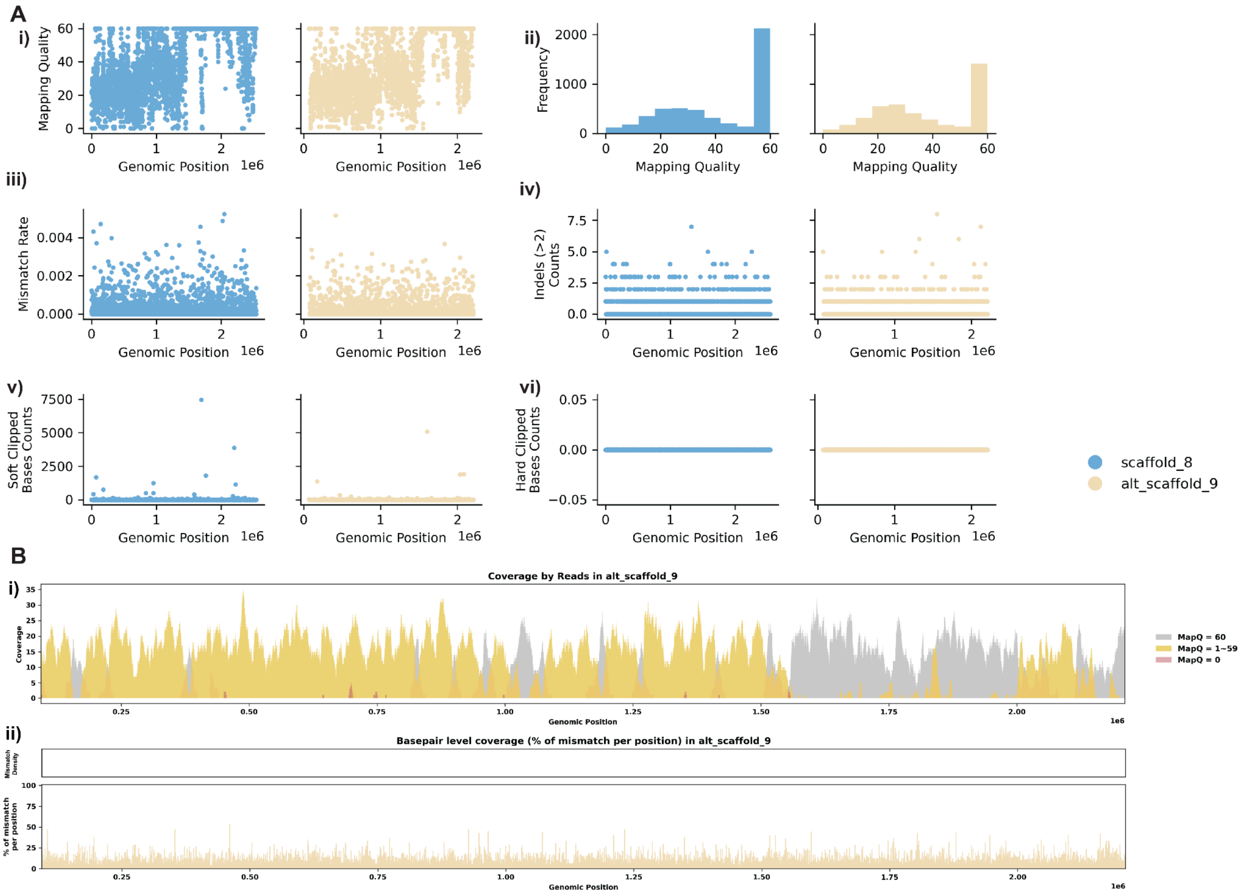

Supplementary Figure 8. Additional Reassembly Analysis of IGH Locus Assembly Errors in *C. volans.* Individual 2 A. Summary statistics of the read alignment are significantly improved. Blue represents the primary assembly and yellow the alternate. i) The plots illustrate the read mapping quality across the IGH loci, ii) read mapping quality distribution, iii) mismatch rates of reads, and iv) number of indels (consecutive length > 2bps), v) count of soft clipped bases, vi) count of hard clipped bases, for both haplotypes across IGH loci. B. A detailed analysis of alignment mismatch in the alternate IGH haplotype includes i) read coverage across the entire IGH loci, color-coded by mapping quality, and ii) *basepair-oriented* mismatch rate, a heatmap above indicating the frequency of high mismatch rate base pairs, with darker colors denoting more frequent occurrences. Light red highlights positions covered by ≥5 reads with an error rate >1%, and purple bars indicate coverage breaks (coverage ≤2).

bioRxiv preprint doi: https://doi.org/10.1101/2024.07.19.604360; this version posted August 2, 2024. The copyright holder for this preprint (which was not certified by peer review) is the author/funder, who has granted bioRxiv a license to display the preprint in perpetuity. It is made available under aCC-BY 4.0 International license.

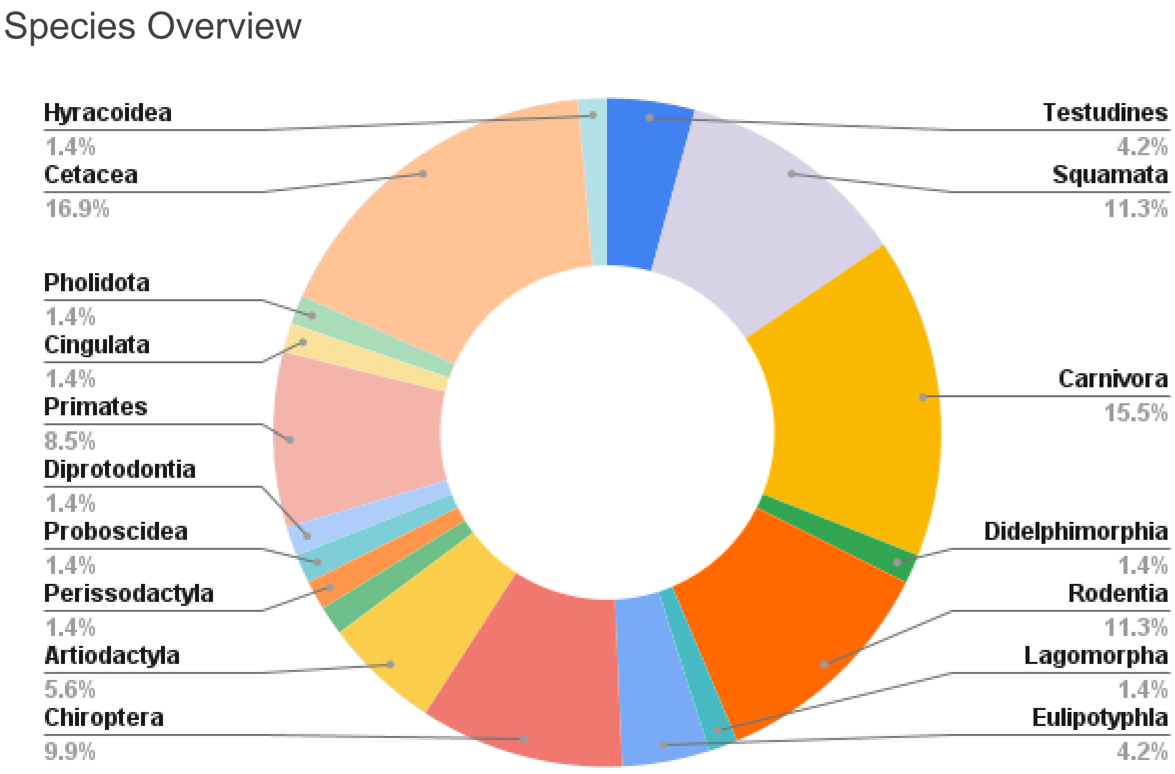

Supplementary Figure 9. Pie chart summarizing the distribution of species by order.

bioRxiv preprint doi: https://doi.org/10.1101/2024.07.19.604360; this version posted August 2, 2024. The copyright holder for this preprint (which was not certified by peer review) is the author/funder, who has granted bioRxiv a license to display the preprint in perpetuity. It is made available under aCC-BY 4.0 International license.

Supplementary Note 1: Comparison to CRAQ and Inspector

We ran CRAQ and Inspector on *C. lupus* individual 1 and *C. lupus* individual 2 to evaluate their effectiveness. In the structural error output BED file provided by Inspector, the IGH loci for both individuals were missing, indicating that Inspector failed to detect structural errors in these regions. Similarly, CRAQ's results showed that the IGH loci for both individuals did not appear in the CSE (Clip-based Structural Errors) and CSH (Clip-based Structural Heterozygosity) outputs. Although CRAQ did identify these errors in its regional error output file, it classified them incorrectly as small-scale errors. This misclassification creates confusion and demonstrates the limitations of both tools in accurately detecting and categorizing errors in the IG loci. This underscores the necessity of developing CloseRead, as relying solely on existing tools like CRAQ and Inspector would have left us unaware of these critical inaccuracies. CloseRead provides a targeted approach to ensure precise detection and classification of errors, addressing the shortcomings of current tools.
