# Supplementary material for: Unveiling Assembly Errors in Immunoglobulin Loci: A Comprehensive Evaluation of Long-read Genome Assemblies Across Vertebrates
Source: bioRxiv. 2024 Jul 23:2024.07.19.604360. Preprint. [Version 1] doi: 10.1101/2024.07.19.604360 (PMC11291089; doi:10.1101/2024.07.19.604360)
Supplement: Supplementary file 1 [file NIHPP2024.02.25.581968V3-supplement-1.pdf]

bioRxiv preprint doi: https://doi.org/10.1101/2024.02.25.581968; this version posted July 26, 2024. The copyright holder for this preprint (which was not certified by peer review) is the author/funder, who has granted bioRxiv a license to display the preprint in perpetuity. It is made available under aCC-BY-NC-ND 4.0 International license.

Supplementary Information

Supplementary Notes

Training and Generation Algorithms

Algorithm 1 Training Algorithm of PocketGen

Input: protein sequences $\mathcal{A} = \boldsymbol{a}_1 \cdots \boldsymbol{a}_{N_s}$ and structures $\{\boldsymbol{x}(\boldsymbol{a}_i)\}_{i=1}^{N_s}$, ligand molecule $\mathcal{M}$, total iteration rounds T.
Initial: Initialize the coordinates of pocket residues $\{\boldsymbol{x}(\boldsymbol{b}_i)\}_{i=1}^{m}$. Initialize the sequences of pocket residues with uniform distributions over 20 residue categories. Initialize the residue/ligand representations $\{\boldsymbol{H}_i\}$.

```
while Model training is not converged do
    # Recycling training
    for t = 1, ··· T do
        Obtain the residue representations {H_i} and updated coordinates {X_i} with the equivariant bilevel graph transformer.
        Predict residue types p_i^t with structure refinement modules.
        if t == T then
            L_seq = Σ_i l_ce(p̂_i, p_i^t).
            L_coord = Σ_i l_huber(X̂_i, X_i^t) + Σ_j l_huber(x̂(v_j), x^t(v_j)).
            L_struct = Σ_{b∈B} l_huber(b̂, b^t) + Σ_{θ∈Θ} l_huber(cos θ̂, cos θ^t)
        end if
    end for
    Minimize λ_seq L_seq + λ_coord L_coord + λ_struct L_struct.
end while
```

Where:
- $\mathcal{L}_{\text{seq}} = \sum_i l_{\text{ce}}(\hat{\boldsymbol{p}}_i, \boldsymbol{p}_i^t)$.
- $\mathcal{L}_{\text{coord}} = \sum_i l_{\text{huber}}(\hat{\boldsymbol{X}}_i, \boldsymbol{X}_i^t) + \sum_j l_{\text{huber}}(\hat{\boldsymbol{x}}(\boldsymbol{v}_j), \boldsymbol{x}^t(\boldsymbol{v}_j))$.
- $\mathcal{L}_{\text{struct}} = \sum_{b \in \mathcal{B}} l_{\text{huber}}(\hat{b}, b^t) + \sum_{\theta \in \Theta} l_{\text{huber}}(\cos\hat{\theta}, \cos\theta^t)$
- Minimize $\lambda_{\text{seq}}\mathcal{L}_{\text{seq}} + \lambda_{\text{coord}}\mathcal{L}_{\text{coord}} + \lambda_{\text{struct}}\mathcal{L}_{\text{struct}}$.

Algorithm 2 Generation Algorithm of PocketGen

Input: protein sequences $\mathcal{A} = \boldsymbol{a}_1 \cdots \boldsymbol{a}_{N_s}$ and structures $\{\boldsymbol{x}(\boldsymbol{a}_i)\}_{i=1}^{N_s}$, ligand molecule $\mathcal{M}$, total iteration rounds T.
Initial: Initialize the coordinates of pocket residues $\{\boldsymbol{x}(\boldsymbol{b}_i)\}_{i=1}^{m}$. Initialize the sequences of pocket residues with uniform distributions over 20 residue categories. Initialize the residue/ligand representations $\{\boldsymbol{H}_i\}$.

```
for t = 1, ..., T do
    Obtain the residue representations {H_i} and updated coordinates {X_i} with the equivariant bilevel graph transformer.
    Predict residue types p_i^t with structure refinement modules.
end for
Map {X_i} to the predicted residue types.
Output designed pockets.
```

---

bioRxiv preprint doi: https://doi.org/10.1101/2024.02.25.581968; this version posted July 26, 2024. The copyright holder for this preprint (which was not certified by peer review) is the author/funder, who has granted bioRxiv a license to display the preprint in perpetuity. It is made available under aCC-BY-NC-ND 4.0 International license.

Loss Functions

In Equation 19, we use Huber loss[91] in $\mathcal{L}_{\text{coord}}$ and $\mathcal{L}_{\text{struct}}$ for the stability of optimization, which are defined as follows:

$$l_{huber}(x,y) = \begin{cases} 0.5\,(x-y)^2, \; if \; |x-y| < \epsilon, \\ \epsilon \cdot (|x-y| - 0.5 \cdot \epsilon), \; else, \end{cases} \tag{20}$$

where x and y represent the predicted and ground-truth coordinates/ bond length/ angles. The Huber loss has the following property: if the L1 norm of $|x-y|$ is smaller than ϵ, it is MSE loss, otherwise it is L1 loss. At the beginning of the model training, the deviation between the predicted and ground-truth coordinates is large, and the L1 term makes the loss less sensitive to outliers than MSE loss. The deviation is small when the training is almost complete, and the MSE loss is applied for further finetuning. In practice, we find that directly using MSE loss sometimes leads to instability at the beginning of the training (e.g., very large gradient norm), while Huber loss makes the training procedure more stable. Following the suggestion of previous works[32,34], we set $\epsilon = 1$ in all our experiments.

bioRxiv preprint doi: https://doi.org/10.1101/2024.02.25.581968; this version posted July 26, 2024. The copyright holder for this preprint (which was not certified by peer review) is the author/funder, who has granted bioRxiv a license to display the preprint in perpetuity. It is made available under aCC-BY-NC-ND 4.0 International license.

Implementation Details of Baseline Methods and Hyperparameter Settings

PocketOptimizer[18] [1] is a physic-based computational protein design method that predicts mutations in the binding pockets of proteins to increase affinity for a specific ligand. We use the latest version, i.e., PocketOptimizer 2.0[18]. There are generally four main steps in PocketOptimizer: structure preparation, flexibility sampling, energy calculations, and computation of design solutions. Specifically for the energy calculations, both packing-related energies and binding-related energies are considered. Following the suggestions in the original paper, we use AMBER ff14S force field[101] for energy computation and the Dunbrack rotamer library[102] for rotamer sampling for PocketOptimizer in our implementation. We fixed the backbone structures following the suggestions of the original paper. As for the output design solutions, we select the top 100 designs identified by PocketOptimizer based on integer linear programming for downstream metric calculations.

DEPACT[17] [2] is a template-matching method that follows a two-step strategy for pocket design. Firstly, it searches the protein-ligand complexes in the template database with similar ligand fragments and constructs a cluster model (a set of pocket residues). The template databases are constructed based on the corresponding training datasets for fair comparisons. Secondly, it grafts the cluster model into the protein pocket with PACMatch. It works by placing residues from the cluster model on protein scaffolds by matching the atoms of residues with atoms of the protein scaffold. The backbone coordinates of the pocket residues are also modified in the process. The qualities of the generated pockets are evaluated and ranked based on a statistical scoring function. We take the top 100 designed pockets for evaluation. The output of DEPACT+PACMatch is complete protein structures with redesigned pockets. In the paper, we only use DEPACT to represent the whole method of DEPACT+PACMatch for conciseness.

RFDiffusion[26] [3] is one of the state-of-the-art method for *de novo* protein backbone generation. It combines the RoseTTAFold structure prediction network with the diffusion probabilistic models (DDPMs) framework. To model the influence of ligand molecules, we use a heuristic attractive-repulsive potential to encourage the formation of pockets with shape complementarity to a target molecule following the suggestions of RFDiffusion[26] and RFDiffusionAA[16]. The residue sequence is further decided with ProteinMPNN[29], and the side-chain conformation is added with Rosetta[99] side-chain packing.

RFDiffusionAA[16] [4] is the latest version of RFDiffusion which combines a residue-based representation of amino acids and atomic representations of all other groups to model protein-small molecules/metals/nucleic acids/covalent modification complexes. Starting from random distributions of amino acid residues surrounding target small molecules, RFDiffusionAA can directly generate the small molecule binding protein backbone. Furthermore, with LigandMPNN[30], the latest version of ProteinMPNN[29], we can assign residue types and predict sidechain conformations considering the protein-ligand interactions. Experiments in RFDiffusionAA[16] show that the generated protein by RFDiffusionAA has better binding affinity than those obtained by RFDiffusion with auxiliary potential.

dyMEAN[25] [5] is an end-to-end full-atom model for E(3)-equivariant antibody design given the epitope and the incomplete sequence of the antibody. Its previous version, MEAN[34], only considers the backbone atoms, while dyMEAN considers the complete atom structure and performs better on downstream tasks. Generally, dyMEAN co-designs antibody sequence and structure via a multi-round progressive full-shot refinement manner, which is more efficient than auto-regressive or diffusion-based approaches. An adaptive multi-channel equivariant encoder is used in dyMEAN, which can process protein residues of variable sizes when considering full atoms. To adapt dyMEAN to our pocket design task, we replace the antigen with the target ligand molecule to provide the context information for pocket generation.

FAIR[24] [6] is our previous method for full atom pocket sequence-structure co-design. FAIR operates in two steps, proceeding in a coarse-to-fine manner (backbone refinement to full atoms refinement, including side chains) for full-atom generation. In FAIR, residue types and atom coordinates are updated using a hierarchical graph transformer composed of a residue-level and atom-level encoder.

[1] https://github.com/Hoecker-Lab/pocketoptimizer
[2] https://github.com/chenyaoxi/DEPACT_PACMatch
[3] https://github.com/RosettaCommons/RFdiffusion
[4] https://github.com/baker-laboratory/rf_diffusion_all_atom
[5] https://github.com/THUNLP-MT/dyMEAN
[6] https://github.com/zaixizhang/FAIR

bioRxiv preprint doi: https://doi.org/10.1101/2024.02.25.581968; this version posted July 26, 2024. The copyright holder for this preprint (which was not certified by peer review) is the author/funder, who has granted bioRxiv a license to display the preprint in perpetuity. It is made available under aCC-BY-NC-ND 4.0 International license.

Proof of E(3)-Equivariance of PocketGen

The E(3)-transformation on the euclidean coordinate $\boldsymbol{x} \in \mathbb{R}^3$ can be represented as: $T_g \cdot \boldsymbol{x} = \boldsymbol{O}\boldsymbol{x} + \boldsymbol{t}$, where $\boldsymbol{O} \in \mathbb{R}^3$ is the orthogonal transformation matrix, $\boldsymbol{t} \in \mathbb{R}^3$ is the translation vector. Implementing T_g on a coordinate matrix $\boldsymbol{X} \in \mathbb{R}^{n\times 3}$ means transforming each coordinate (i.e., each row) with T_g. PocketGen has the desirable property of **E(3)-equivariance** as follow:

Theorem 1. *Denote the E(3)-transformation as T_g and the generative process of PocketGen as $\{(\boldsymbol{b}_i, \boldsymbol{x}(\boldsymbol{b}_i))\}_{i=1}^m = p_\theta(\mathcal{A}\setminus\mathcal{B}, \mathcal{M})$, where $\{(\boldsymbol{b}_i, \boldsymbol{x}(\boldsymbol{b}_i))\}_{i=1}^m$ indicates the designed protein pocket seqeuce and structure. We have $\{(\boldsymbol{b}_i, T_g \cdot \boldsymbol{x}(\boldsymbol{b}_i))\}_{i=1}^m = p_\theta(T_g \cdot (\mathcal{A}\setminus\mathcal{B}), T_g \cdot \mathcal{M})$. Here, $T_g \cdot (\mathcal{A}\setminus\mathcal{B})$ and $T_g \cdot \mathcal{M}$ denote applying E(3)-transformation on the protein structures except for the pocket region and the molecular structure respectively.*

Then we prove the E(3)-equivariance of each module in PocketGen as follows.

Lemma 1. *Denote the bilevel attention module as $\{\boldsymbol{H}_i', \boldsymbol{X}_i'\} = \mathrm{Att}(\{\boldsymbol{H}_i, \boldsymbol{X}_i\})$, then it is E(3)-equivariant. Namely, for any E(3)-transformation T_g, we have $\{\boldsymbol{H}_i', T_g \cdot \boldsymbol{X}_i'\} = \mathrm{Att}(\{\boldsymbol{H}_i, T_g \cdot \boldsymbol{X}_i\})$.*

Proof. The key to the proof of Lemma 1 is to prove that the propagation in Eq. 2-9 is E(3)-invariant on $\boldsymbol{H}_i$ and E(3)-equivariant on $\boldsymbol{X}_i$. The correlation $\boldsymbol{R}_{ij}$ between block i and block j in Eq. 3 is E(3)-invariant because all the inputs, including the query $\boldsymbol{Q}_i$, the key $\boldsymbol{K}_j$, and the distance matrices $\boldsymbol{D}_{ij}$, are not influenced by the geometric transformation T_g. Therefore, we can immediately derive that the atom-level attention $\boldsymbol{\alpha}_{ij}$ in Eq. 4 is E(3)-invariant. Similarly, the residue/ligand-level attention β_{ij} in Eq. 6 is E(3)-invariant because it only operates on r_{ij} in Eq. 5 which aggregates $\boldsymbol{\alpha}_{ij}$. Finally, we can derive the E(3)-invariance on $\boldsymbol{H}$ and the E(3)-equivariance on $\boldsymbol{X}$ (ligand or pocket residues) as below:

$$
\begin{aligned}
\boldsymbol{H}_i'[p] &= \boldsymbol{H}_i[p] + \sum_{j\in\mathcal{N}(i)} \beta_{ij}\phi_h(\boldsymbol{\alpha}_{ij}[p]\cdot\boldsymbol{V}_j),\\
T_g\cdot\boldsymbol{X}_i'[p] &= T_g\cdot(\boldsymbol{X}_i[p] + \sum_{j\in\mathcal{N}(i)} \beta_{ij}\left(\boldsymbol{\alpha}_{ij}[p]\odot\phi_x(\boldsymbol{Q}_i[p]||\boldsymbol{K}_j||\mathrm{RBF}(\boldsymbol{D}_{ij}[p]))\right)\cdot\boldsymbol{X}_{ij}[p])\\
&= \boldsymbol{O}(\boldsymbol{X}_i[p] + \sum_{j\in\mathcal{N}(i)} \beta_{ij}\left(\boldsymbol{\alpha}_{ij}[p]\odot\phi_x(\boldsymbol{Q}_i[p]||\boldsymbol{K}_j||\mathrm{RBF}(\boldsymbol{D}_{ij}[p]))\right)\cdot\boldsymbol{X}_{ij}[p]) + \boldsymbol{t}\\
&= (\boldsymbol{O}\boldsymbol{X}_i[p]+\boldsymbol{t}) + \sum_{j\in\mathcal{N}(i)} \beta_{ij}\left(\boldsymbol{\alpha}_{ij}[p]\odot\phi_x(\boldsymbol{Q}_i[p]||\boldsymbol{K}_j||\mathrm{RBF}(\boldsymbol{D}_{ij}[p]))\right)\cdot\begin{bmatrix}\boldsymbol{O}(\boldsymbol{X}_i[p]-\boldsymbol{X}_j[1])\\ \vdots \\ \boldsymbol{O}(\boldsymbol{X}_i[p]-\boldsymbol{X}_j[n_j])\end{bmatrix}\\
&= (\boldsymbol{O}\boldsymbol{X}_i[p]+\boldsymbol{t}) + \sum_{j\in\mathcal{N}(i)} \beta_{ij}\left(\boldsymbol{\alpha}_{ij}[p]\odot\phi_x(\boldsymbol{Q}_i[p]||\boldsymbol{K}_j||\mathrm{RBF}(\boldsymbol{D}_{ij}[p]))\right)\cdot\begin{bmatrix}\boldsymbol{O}\boldsymbol{X}_i[p]+\boldsymbol{t}-(\boldsymbol{O}\boldsymbol{X}_j[1]+\boldsymbol{t})\\ \vdots \\ \boldsymbol{O}\boldsymbol{X}_i[p]+\boldsymbol{t}-(\boldsymbol{O}\boldsymbol{X}_j[n_j]+\boldsymbol{t})\end{bmatrix}\\
&= T_g\cdot\boldsymbol{X}_i[p] + \sum_{j\in\mathcal{N}(i)} \beta_{ij}\left(\boldsymbol{\alpha}_{ij}[p]\odot\phi_x(\boldsymbol{Q}_i[p]||\boldsymbol{K}_j||\mathrm{RBF}(\boldsymbol{D}_{ij}[p]))\right)\cdot\begin{bmatrix}T_g\cdot\boldsymbol{X}_i[p]-T_g\cdot\boldsymbol{X}_j[1]\\ \vdots \\ T_g\cdot\boldsymbol{X}_i[p]-T_g\cdot\boldsymbol{X}_j[n_j]\end{bmatrix},
\end{aligned}
$$

which concludes the proof of Lemma 1. □

Lemma 2. *Denote the equivariant feed-forward network as as $\{\boldsymbol{H}_i', \boldsymbol{X}_i'\} = \mathrm{FFN}(\{\boldsymbol{H}_i, \boldsymbol{X}_i\})$, then it is E(3)-equivariant. Namely, for any E(3)-transformation T_g, we have $\{\boldsymbol{H}_i', T_g \cdot \boldsymbol{X}_i'\} = \mathrm{FFN}(\{\boldsymbol{H}_i, T_g \cdot \boldsymbol{X}_i\})$.*

Proof. The proof of Lemma 2 focuses on the single-atom updates in Eq. 10-13. First, it is easy to obtain the E(3)-equivariance of the centroid in Eq. 10:

$$T_g\cdot\boldsymbol{x}_c = T_g\cdot\mathrm{centroid}(\boldsymbol{X}_i) = \mathrm{centroid}(T_g\cdot\boldsymbol{X}_i).$$

Then we have the following equation on the relative coordinate $\Delta\boldsymbol{x}$ in Eq. 11:

$$\boldsymbol{O}\Delta\boldsymbol{x}_p = (\boldsymbol{O}\boldsymbol{X}_i[p]+\boldsymbol{t}) - (\boldsymbol{O}\boldsymbol{x}_c+\boldsymbol{t}) = T_g\cdot\boldsymbol{X}_i[p] - T_g\cdot\boldsymbol{x}_c.$$

Then we can obtain the E(3)-invariance of $\boldsymbol{r}_p$ in Eq. 11:

$$\boldsymbol{r}_p = \mathrm{RBF}(\|\Delta\boldsymbol{x}_p\|_2) = \mathrm{RBF}(\|\boldsymbol{X}_i[p]-\boldsymbol{x}_c\|_2) = \mathrm{RBF}(\|(\boldsymbol{O}\boldsymbol{X}_i[p]+\boldsymbol{t})-(\boldsymbol{O}\boldsymbol{x}_c+\boldsymbol{t})\|_2) = \mathrm{RBF}(\|T_g\cdot\boldsymbol{X}_i[p]-T_g\cdot\boldsymbol{x}_c\|_2).$$

bioRxiv preprint doi: https://doi.org/10.1101/2024.02.25.581968; this version posted July 26, 2024. The copyright holder for this preprint (which was not certified by peer review) is the author/funder, who has granted bioRxiv a license to display the preprint in perpetuity. It is made available under aCC-BY-NC-ND 4.0 International license.

Finally we can derive the E(3)-invariance on $\boldsymbol{H}'[p]$ and the E(3)-equivariance on $\boldsymbol{X}_i'[p]$:

$$
\begin{aligned}
\boldsymbol{H}'[p] &= \boldsymbol{H}[p] + \sigma_h(\boldsymbol{H}_i[p], \boldsymbol{h}_c, \boldsymbol{r}_p), \\
T_g \cdot \boldsymbol{X}_i'[p] &= T_g \cdot (\boldsymbol{X}_i[p] + \Delta\boldsymbol{x}_p \sigma_x(\boldsymbol{H}_i[p], \boldsymbol{h}_c, \boldsymbol{r}_p)) \\
&= \boldsymbol{O}(\boldsymbol{X}_i[p] + \Delta\boldsymbol{x}_p \sigma_x(\boldsymbol{H}_i[p], \boldsymbol{h}_c, \boldsymbol{r}_p)) + \boldsymbol{t} \\
&= \boldsymbol{O}\boldsymbol{X}_i[p] + \boldsymbol{t} + \boldsymbol{O}\Delta\boldsymbol{x}_p \sigma_x(\boldsymbol{H}_i[p], \boldsymbol{h}_c, \boldsymbol{r}_p) \\
&= T_g \cdot \boldsymbol{X}_i[p] + (T_g \cdot \boldsymbol{X}_i[p] - T_g \cdot \boldsymbol{x}_c)\sigma_x(\boldsymbol{H}_i[p], \boldsymbol{h}_c, \boldsymbol{r}_p) \\
&= T_g \cdot \boldsymbol{X}_i[p] + (T_g \cdot \boldsymbol{X}_i[p] - \text{centroid}(T_g \cdot \boldsymbol{X}_i))\sigma_x(\boldsymbol{H}_i[p], \boldsymbol{h}_c, \boldsymbol{r}_p),
\end{aligned}
$$

which concludes the proof of Lemma 2 □

The sequence refinement module with the pretrained protein language models and adapters operates on the scaler residue representations unaffected by the geometric transformation T_g. To sum up, with Lemma 1-2 at hand, it is obvious to deduce the E(3)-equivariance of the PocketGen.

bioRxiv preprint doi: https://doi.org/10.1101/2024.02.25.581968; this version posted July 26, 2024. The copyright holder for this preprint (which was not certified by peer review) is the author/funder, who has granted bioRxiv a license to display the preprint in perpetuity. It is made available under aCC-BY-NC-ND 4.0 International license.

Supplementary Figures

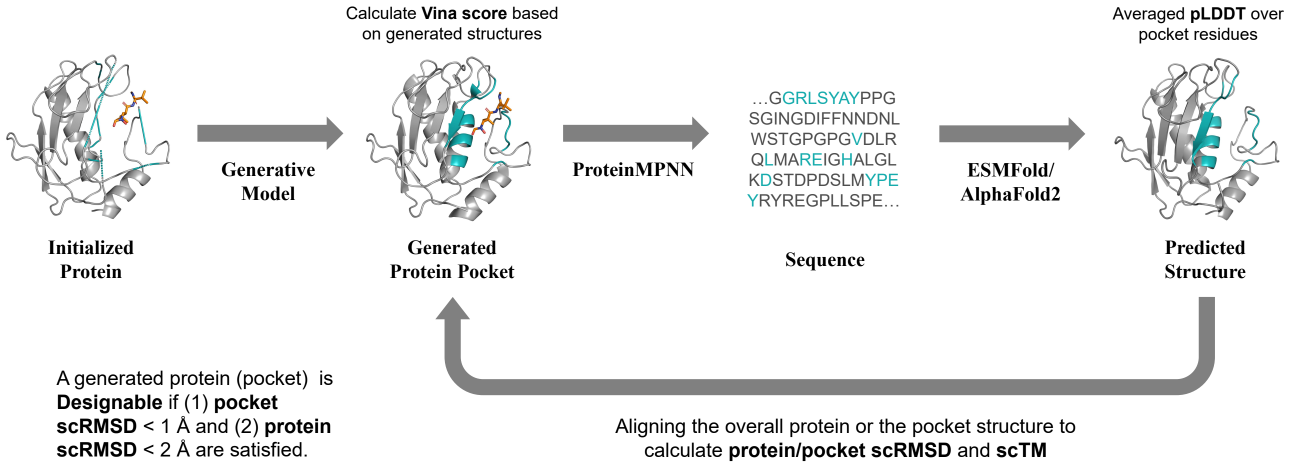

Figure S1. Schematic of computing metrics with respect to the generated pocket structures. black parts refer to the pocket, while the others are gray. The generative model refers to PocketGen or baseline methods leveraged to generate the protein pocket. The Vina score is calculated based on the generated protein-ligand complex. To calculate self-consistency scores, we first use ProteinMPNN to derive the residue sequence and then use ESMFold/AlphaFold2 to predict the structure. By aligning the predicted structure with the generated structure, we can obtain the protein/pocket scRMSD. Similarly, we can obtain the scTM score of the protein structure. We also report the averaged pLDDT of the pocket residues from ESMFold.

bioRxiv preprint doi: https://doi.org/10.1101/2024.02.25.581968; this version posted July 26, 2024. The copyright holder for this preprint (which was not certified by peer review) is the author/funder, who has granted bioRxiv a license to display the preprint in perpetuity. It is made available under aCC-BY-NC-ND 4.0 International license.

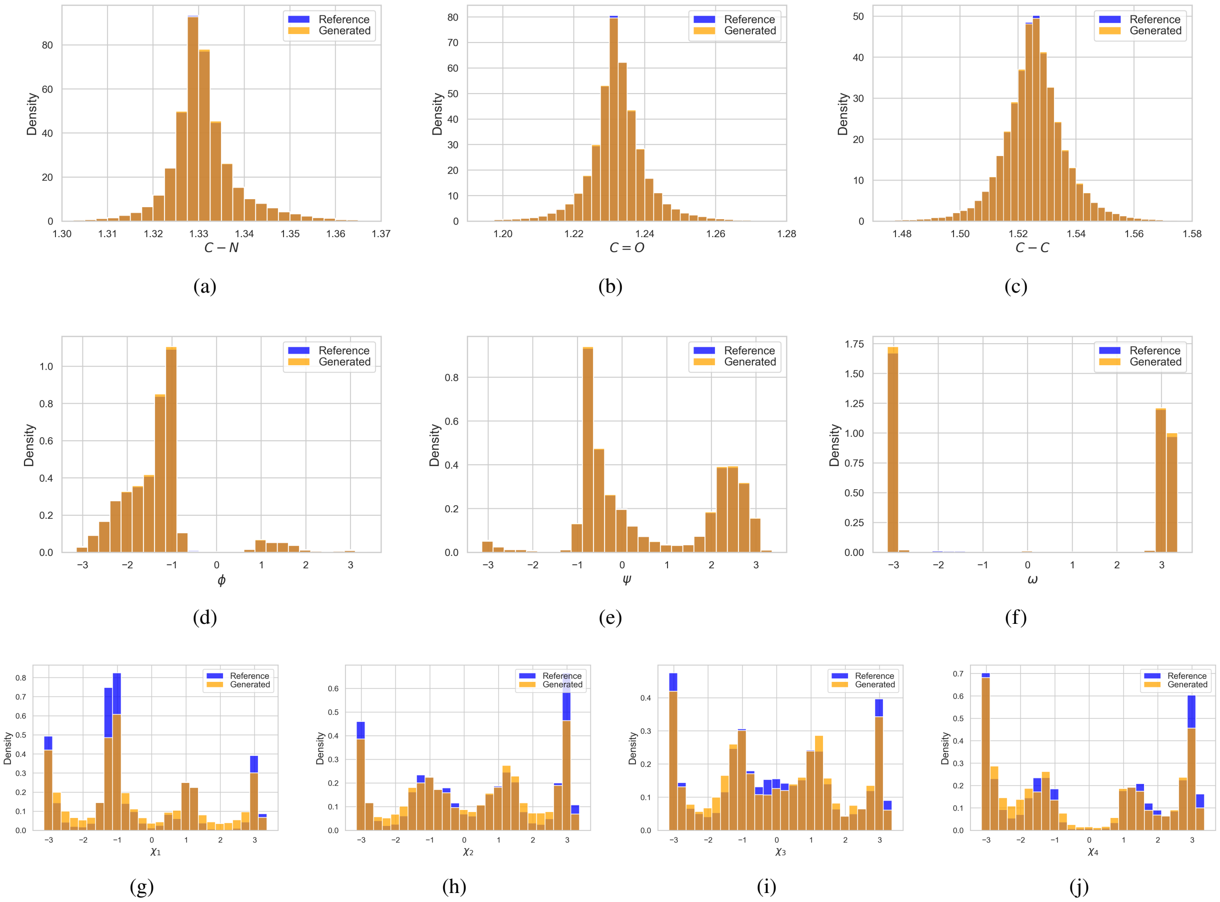

Figure S2. The bond length and dihedral angle distributions of the generated pockets and the reference (training set of CrossDocked dataset).

bioRxiv preprint doi: https://doi.org/10.1101/2024.02.25.581968; this version posted July 26, 2024. The copyright holder for this preprint (which was not certified by peer review) is the author/funder, who has granted bioRxiv a license to display the preprint in perpetuity. It is made available under aCC-BY-NC-ND 4.0 International license.

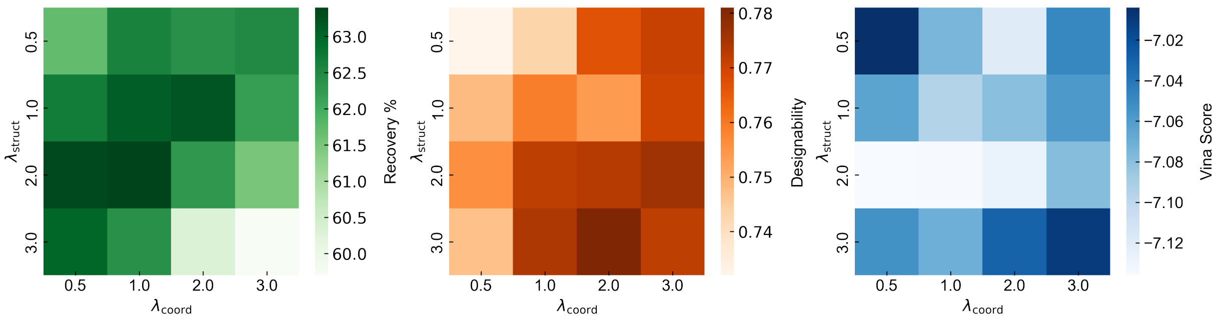

(a) AAR (b) Designability (c) Vina Score

Figure S3. Hyperparameter Analysis. Influence of hyperparameters (loss weights $\mathcal{L}_{\text{coord}}$ and $\mathcal{L}_{\text{struct}}$) on PocketGen's performance (AAR, Designability, and Vina Score) on the CrossDocked dataset. We perform a grid search over $\{0.5, 1.0, 2.0, 3.0\}$ and choose these hyperparameters based on the validation performance to select the specific parameter values. In the default setting, we set λ_{coord} to 1.0 and λ_{struct} to 2.0.

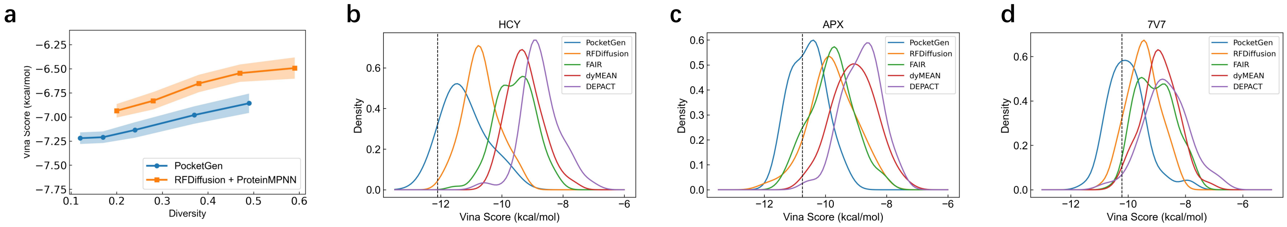

Figure S4. Additional results with RFDiffusion. a, The trade-off between quality (measured by Vina score) and diversity (1-average pairwise sequence similarity) of PocketGen. **b, c, d,** The pocket binding affinity distributions of PocketGen and baseline methods for three target molecules (HCY, APX, and 7V7, respectively).

bioRxiv preprint doi: https://doi.org/10.1101/2024.02.25.581968; this version posted July 26, 2024. The copyright holder for this preprint (which was not certified by peer review) is the author/funder, who has granted bioRxiv a license to display the preprint in perpetuity. It is made available under aCC-BY-NC-ND 4.0 International license.

Supplementary Tables

Table S1. Benchmarking PocketGen and other approaches for pocket generation on two datasets. Reported are average and standard deviation values across three independent runs (random seeds on the same dataset). The best results are bolded.

Model	CrossDocked			Binding MOAD		
	AAR (↑)	Designability (↑)	Vina (↓)	AAR (↑)	Designability (↑)	Vina (↓)
Test set	-	0.77	-7.016	-	0.79	-8.076
DEPACT	31.52±3.26%	0.68±0.04	-6.632±0.18	35.30±2.19%	0.67±0.06	-7.571±0.15
dyMEAN	38.71±2.16%	0.71±0.03	-6.855±0.06	41.22±1.40%	0.70±0.03	-7.675±0.09
FAIR	40.16±1.17%	0.73±0.02	-7.015±0.12	43.68±0.92%	0.72±0.05	-7.930±0.15
RFDiffusion	46.57±2.07%	0.74±0.01	-6.936±0.07	45.31±2.73%	0.75±0.05	-7.942±0.14
RFDiffusionAA	50.85±1.85%	0.75±0.03	-7.012±0.09	49.09±2.49%	0.78±0.03	-8.020±0.11
PocketGen	**63.40±1.64%**	**0.77±0.02**	**-7.135±0.08**	**64.43±2.35%**	**0.80±0.04**	**-8.112±0.14**

bioRxiv preprint doi: https://doi.org/10.1101/2024.02.25.581968; this version posted July 26, 2024. The copyright holder for this preprint (which was not certified by peer review) is the author/funder, who has granted bioRxiv a license to display the preprint in perpetuity. It is made available under aCC-BY-NC-ND 4.0 International license.

Table S2. The top 1/3/5/10 generated protein pocket (ranked by Vina score) with designability on the CrossDocked dataset. The success rate measures the percentage of protein that the model can generate pockets with higher affinity than the reference ones in the datasets. We report the average plDDT of the predicted pocket, the scRMSD of the pocket backbone coordinates, and the change of the scTM score of the whole protein. ESMFold means the scores are calculated with AlphaFold2 as the folding tool. The plDDT, scRMSD, and ΔscTM for PocketOpt are not reported, as PocketOpt keeps protein backbone structures fixed. We use to mark the results of affinity-related metrics, for pocket-structure related metrics, and for whole protein structure metrics. We report the means and standard deviations over three independent runs with random seeds.

	DEPACT	dyMEAN	FAIR	RFDiffusion	RFAA	PocketGen
Top-1 generated protein pocket						
pLDDT (ESMFold) (↑)	82.130±0.187	83.327±0.206	83.254±0.412	84.559±0.230	86.346±0.238	**87.065**±0.180
scRMSD (ESMFold) (↓)	0.705±0.023	0.703±0.024	0.680±0.019	0.676±0.017	0.654±0.011	**0.642**±0.008
ΔscTM (ESMFold) (↑)	-0.009±0.002	-0.004±0.003	-0.011±0.004	0.014±0.005	0.021±0.003	**0.030**±0.004
ΔscTM (ESMFold+co) (↑)	-0.015±0.005	-0.016±0.003	-0.023±0.005	-	-	**0.014**±0.003
Top-3 generated protein pockets						
pLDDT (ESMFold) (↑)	81.991±0.303	82.825±0.218	83.145±0.297	84.636±0.186	86.224±0.190	**86.450**±0.075
scRMSD (ESMFold) (↓)	0.706±0.025	0.724±0.024	0.685±0.017	0.679±0.014	**0.653**±0.012	0.655±0.007
ΔscTM (ESMFold) (↑)	-0.014±0.003	-0.010±0.004	-0.013±0.005	0.019 ±0.004	0.020±0.003	**0.024** ±0.001
ΔscTM (ESMFold+co) (↑)	-0.016±0.003	-0.022±0.002	-0.026±0.003	-	-	**0.012**±0.002
Top-5 generated protein pockets						
pLDDT (ESMFold) (↑)	82.070±0.276	82.910±0.231	83.168±0.208	84.320±0.219	85.735±0.087	**86.414**±0.110
scRMSD (ESMFold) (↓)	0.717±0.019	0.725±0.014	0.690±0.012	0.680±0.012	0.656±0.006	**0.654**±0.010
ΔscTM (ESMFold) (↑)	-0.018±0.004	-0.007 ±0.002	-0.024±0.002	0.016±0.001	0.019±0.001	**0.027**±0.001
ΔscTM (ESMFold+co) (↑)	-0.014±0.004	-0.023±0.003	-0.027±0.002	-	-	**0.015**±0.002
Top-10 generated protein pockets						
pLDDT (ESMFold) (↑)	81.580±0.232	82.771±0.203	83.048±0.165	84.234±0.195	85.377±0.142	**86.090**±0.124
scRMSD (ESMFold) (↓)	0.710±0.014	0.734±0.013	0.705±0.013	0.684±0.012	0.672±0.007	**0.657**±0.005
ΔscTM (ESMFold) (↑)	-0.025±0.002	-0.014±0.001	-0.026±0.002	0.014±0.003	0.019±0.000	**0.022**±0.001
ΔscTM (ESMFold+co) (↑)	-0.015±0.003	-0.022±0.002	-0.026±0.003	-	-	**0.013**±0.001

bioRxiv preprint doi: https://doi.org/10.1101/2024.02.25.581968; this version posted July 26, 2024. The copyright holder for this preprint (which was not certified by peer review) is the author/funder, who has granted bioRxiv a license to display the preprint in perpetuity. It is made available under aCC-BY-NC-ND 4.0 International license.

Table S3. The top 1/3/5/10 generated protein pocket (ranked by Vina score) with designability on the Binding MOAD dataset. Besides Vina score, we also calculate MM-GBSA to evaluate the binding affinity. The success rate measures the percentage of protein that the model can generate pockets with higher affinity than the reference ones in the datasets. We report the average plDDT of the predicted pocket, the scRMSD of the pocket backbone coordinates, and the change of the scTM score of the whole protein. co indicates codesign, where codesign methods directly use the designed sequence for consistency calculation. The plDDT, scRMSD, and ΔscTM for PocketOpt are not reported, as PocketOpt keeps protein backbone structures fixed. We use ▢ to mark the results of affinity-related metrics, ▢ for pocket-structure related metrics, and ▢ for whole protein structure metrics. We report the means and standard deviations over three independent runs with random seeds.

Methods:	PocketOpt	DEPACT	dyMEAN	FAIR	RFDiffusion	RFAA	PocketGen
Top-1 generated protein pocket							
Vina score (↓)	-9.828±0.120	-9.216±0.071	-9.352±0.082	-9.530±0.064	-9.901±0.055	-10.120±0.063	**-10.322**±0.057
MM-GBSA (↓)	-62.293±0.819	-55.180±0.906	-55.504±0.628	-59.851±0.723	-63.440±0.836	-64.305±1.04	**-67.862**±0.618
Success Rate (↑)	0.891±0.024	0.784±0.045	0.779±0.02	0.841±0.033	0.905±0.030	0.923±0.027	**0.952**±0.016
pLDDT (ESMFold) (↑)	-	83.145±0.426	83.280±0.247	83.536±0.217	85.068±0.335	**87.232**±0.314	87.106±0.224
scRMSD (ESMFold) (↓)	-	0.642±0.024	0.622±0.017	0.620±0.025	0.581±0.018	**0.572**±0.016	0.575±0.013
ΔscTM (ESMFold) (↑)	-	-0.004±0.001	-0.001±0.002	0.008±0.003	0.025±0.005	**0.031**±0.005	0.030±0.004
ΔscTM (ESMFold+co) (↑)	-	-0.025±0.003	-0.019±0.004	-0.008±0.005	-	-	0.004±0.003
Top-3 generated protein pocket							
Vina score (↓)	-9.403±0.102	-8.876±0.064	-8.971±0.076	-9.234±0.050	-9.589±0.048	-9.634±0.045	**-10.135**±0.039
MM-GBSA (↓)	-58.204±0.739	-51.255±0.660	-52.371±0.540	-54.946±0.632	-57.235±0.803	-60.801±0.829	**-64.108**±0.421
pLDDT (↑)	-	82.769±0.370	82.131±0.263	82.826±0.207	84.912±0.328	85.920±0.301	**86.535**±0.220
scRMSD (ESMFold)(↓)	-	0.645±0.023	0.620±0.015	0.596±0.014	0.580±0.016	0.574±0.015	**0.573**±0.011
ΔscTM (ESMFold) (↑)	-	-0.009±0.002	-0.004±0.003	0.004±0.002	0.020±0.005	0.025±0.004	**0.029**±0.003
ΔscTM (ESMFold+co) (↑)	-	-0.027±0.002	-0.020±0.003	-0.007±0.002	-	-	**0.005**±0.002
Top-5 generated protein pocket							
Vina score (↓)	-9.260±0.091	-8.759±0.050	-8.842±0.050	-9.195±0.043	-9.478±0.045	-9.569±0.035	**-9.950**±0.030
MM-GBSA (↓)	-55.728±0.536	-49.204±0.745	-50.289±0.431	-52.152±0.577	-55.490±0.830	-56.306±0.687	**-62.970**±0.412
pLDDT (ESMFold) (↑)	-	81.939±0.261	81.915±0.163	82.346±0.219	85.150±0.322	85.830±0.205	**86.438**±0.160
scRMSD (ESMFold) (↓)	-	0.639±0.015	0.628±0.014	0.612±0.015	0.587±0.010	0.583±0.011	**0.581**±0.008
ΔscTM (ESMFold) (↑)	-	-0.007±0.001	-0.002±0.002	0.003±0.002	0.024±0.003	0.024±0.003	**0.026**±0.001
ΔscTM (ESMFold+co) (↑)	-	-0.027±0.004	-0.023±0.001	-0.005±0.002	-	-	**0.005**±0.002
Top-10 generated protein pocket							
Vina score (↓)	-8.981±0.087	-8.632±0.056	-8.690±0.051	-8.827±0.063	-9.268±0.042	-9.320±0.054	**-9.630**±0.037
MM-GBSA (↓)	-51.337±0.505	-45.480±0.519	-47.804±0.420	-48.203±0.625	-53.660±0.522	-55.763±0.643	**-60.106**±0.284
pLDDT (ESMFold) (↑)	-	81.632±0.270	81.774±0.264	82.739±0.340	84.582±0.191	85.337±0.229	**86.456**±0.079
scRMSD (ESMFold) (↓)	-	0.644±0.012	0.626±0.014	0.592±0.013	0.589±0.014	0.586±0.007	**0.584**±0.005
ΔscTM (ESMFold) (↑)	-	-0.003±0.002	-0.006±0.001	0.006±0.003	0.012±0.002	0.018±0.002	**0.027**±0.001
ΔscTM (ESMFold+co) (↑)	-	-0.029±0.002	-0.021±0.001	-0.008±0.002	-	-	**0.006**±0.003

Table S4. Substructure analysis of the generated molecules. We consider three covalent bonds in the backbone (C-N, C=O, and C-C), three conventional dihedral angles in the backbone (ϕ,ψ,ω[58]), and four dihedral angles in the side chains ($\chi_1,\chi_2,\chi_3,\chi_4$[59]). The KL divergence of the bond lengths and dihedral angles between the training set (CrossDocked) and the generated pockets are calculated following previous works[60,61]. Since PocketOpt sets the pocket backbone fixed, the corresponding backbone metrics are not calculated.

Methods	C-N	C=O	C-C	ϕ	ψ	ω	χ_1	χ_2	χ_3	χ_4
PocketOpt	-	-	-	-	-	-	**0.140**	0.098	0.071	0.104
DEPACT	0.010	0.011	0.009	0.033	0.032	0.097	0.190	0.126	0.109	0.148
dyMEAN	0.012	0.012	0.022	0.036	0.017	0.078	0.224	0.187	0.130	0.125
FAIR	0.014	0.011	0.020	0.025	0.024	0.080	0.235	0.067	0.110	0.167
RFDiffusion	0.009	0.006	0.006	0.027	0.016	0.066	0.160	0.092	0.073	**0.060**
RFDiffusionAA	0.008	0.007	**0.005**	**0.025**	0.014	**0.062**	0.159	0.087	0.071	0.064
PocketGen	**0.006**	**0.004**	**0.005**	0.029	**0.013**	0.077	0.158	**0.053**	**0.065**	0.097

bioRxiv preprint doi: https://doi.org/10.1101/2024.02.25.581968; this version posted July 26, 2024. The copyright holder for this preprint (which was not certified by peer review) is the author/funder, who has granted bioRxiv a license to display the preprint in perpetuity. It is made available under aCC-BY-NC-ND 4.0 International license.

Table S5. Ablations studies of PocketGen. **PocketGen w/o ligand update** indicates the ligand structure is fixed during the generation of pockets by PocketGen. **PocketGen w/o residue-level attention** denotes only the basic atom-level attention performed in the bilevel attention module. **PocketGen w/o pLM** means no pretrained protein language model is leveraged for sequence refinement. Moreover, **PocketGen w/ EGNN/GVP/GMN** uses EGNN[67], GVP[68], and GMN[69] respectively to replace the bilevel graph transformer in PocketGen as the structural encoder for comparison. We report the means and standard deviations over three different runs (%). The best results are bolded.

Model	CrossDocked			Binding MOAD		
	AAR (↑)	Designability (↑)	Vina (↓)	AAR (↑)	Designability (↑)	Vina (↓)
PocketGen w/o ligand update	59.20±1.56%	0.75±0.03	-6.838±0.10	59.61±2.13%	0.77±0.05	-7.865±0.12
PocketGen w/o residue-level attention	58.19±1.78%	0.66±0.04	-6.924±0.08	57.30±2.28%	0.67±0.04	-7.805±0.10
PocketGen w/o pLM	42.70±1.45%	0.71±0.05	-6.894±0.07	44.23±1.31%	0.69±0.06	-7.787±0.12
PocketGen w/ EGNN encoder	58.85±1.21%	0.71±0.02	-6.916±0.03	58.64±1.14%	0.72±0.06	-7.804±0.11
PocketGen w/ GVP encoder	61.10±0.90%	0.72±0.04	-6.930±0.08	59.97±1.40%	0.75±0.05	-7.883±0.10
PocketGen w/ GMN encoder	60.49±1.33%	0.68±0.03	-6.947±0.05	59.82±1.67%	0.74±0.04	-7.869±0.08
PocketGen	**63.40±1.64%**	**0.77±0.02**	**-7.135±0.08**	**64.43±2.35%**	**0.80±0.04**	**-8.112±0.14**

bioRxiv preprint doi: https://doi.org/10.1101/2024.02.25.581968; this version posted July 26, 2024. The copyright holder for this preprint (which was not certified by peer review) is the author/funder, who has granted bioRxiv a license to display the preprint in perpetuity. It is made available under aCC-BY-NC-ND 4.0 International license.

Table S6. Hyperparameters for the baselines and our PocketGen.

hyperparameter	value	description
		PocketOptimizer
n_confs	50	The number of sampled ligand conformers.
n_poses	10000	The max number of ligand poses to sample.
ligand_scaling	1.0	The ligand scaling factor.
vdw_filter_thresh	100	The energy threshold for filtering rotamers (kcal/mol).
		DEPACT
interaction_threshold	-1.0	The threshold of ligand-subpocket interaction score for filtering.
mathcing_threshold	1.5 Å	The RMSD cutoff for finding geometrically consistent ways of placing the seeding residues.
num_CCSS	1000	The number of common chemical substructure (CCSS).
weight_seed	100	The weight of seeding residues in the scoring function.
		FAIR
hidden_size	128	Size of the hidden states in its hierarchical message passing network (MPN).
num_heads	4	Number of attention heads.
n_atom_layers	6	Number of atom-level layers in the MPN.
n_residue_layers	2	Number of residue-level layers in the MPN.
k_atom_neighbors	24	Number of neighbors for each node in the atom KNN graph.
k_residue_neighbors	8	Number of neighbors for each node in the residue KNN graph.
n_backbone_iter	5	Number of iterations in backbone refinement.
n_fullatom_iter	10	Number of iterations in full atom refinement.
		RFDiffusion
potential_scale	1.0	Scale of the auxiliary substrate contact potential.
temp_ProteinMPNN	0.1	The temperature in ProteinMPNN for sequence inference.
n_steps	50	Number of the diffusion steps.
		RFDiffusionAA
temp_ProteinMPNN	0.1	The temperature in LigandMPNN for sequence inference.
n_steps	50	Number of the diffusion steps.
		dyMEAN
embed_size	64	Size of the residue type embedding and the position number embedding.
hidden_size	128	Size of the hidden states in the MPN
n_layers	3	Number of layers in the MPN
n_iter	3	Number of iterations in the progressive full-shot decoding.
k_neighbors	9	Number of neighbors for each node in the KNN graph.
d	16	Size of the attribute vector of each channel (equal to the size of the atom type
		PocketGen
hidden_size	128	Size of the hidden states in the MPN
n_layers	4	Number of layers in the MPN
num_heads	4	Number of attention heads.
n_iter	3	Number of refinement rounds.
k_sparse	3	Number of elements to keep in the sparse attention.
k_neighbors	8	Number of neighbors for each node in the KNN graph.
